# Modeling Alkylcopper Decomposition Pathway: Deciphering Pre‐Nucleation Processes for Metallic Cu Nanoparticles Synthesis

**DOI:** 10.1002/chem.71366

**Published:** 2026-07-12

**Authors:** Marina Descoubes, Hélène Gérard

**Affiliations:** ^1^ Laboratoire De Chimie Théorique (LCT) Sorbonne Université CNRS Paris France

**Keywords:** C—H activation, copper, DFT, pre‐nucleation, reaction mechanism

## Abstract

This study investigates the thermal decomposition mechanism of ethylcopper through density functional theory (DFT) calculations, focusing on the pathways leading to the formation of ethane. We emphasize the importance of aggregation and Cu—Cu bond formation and evaluate the autocatalytic effect of Cu^(0)^ atoms in these mechanisms. The retained reactions pathways go through a ẞ‐H elimination step exhibiting a relatively low activation barrier but resulting in metastable hydrido‐copper complexes. It is followed by the reduction step itself which is found to be the rate determining step. Formation of trimers from the start or in the course of the reaction is found to play a major role in the thermal decomposition process of ethylcopper. Together, these insights are confronted to experimental data from the literature and provide a comprehensive understanding of the thermodynamic and kinetic aspect of these pre‐nucleation processes.

## Graphical abstract

Computational study reveals a two‐step decomposition of alkylcopper complexes into (partially) reduced Cu clusters, proposed as prenucleation species for Cu^(0)^ nanoparticle synthesis. Cu‐hydride intermediates and multinuclear centers lower reaction barriers.

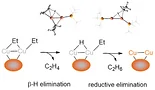

## 1 Introduction

Copper nanoparticles (Cu NPs) are well known and widely used for their catalytic [1] and plasmonic [2, 3] properties. In both cases, the control of size and shape is essential for the control of the physicochemical properties [4, 5, 6, 7]. Understanding the nature of the pre‐nucleation intermediates and the chemical reactivity at stake in the nucleation process is attractive as it could pave the way to rational design of the synthetic process [8]. In particular, a correlation was evidenced between the kinetic of the monomer formation and the final shape of the NPs. However, in the case Cu NPs, the harsh reaction conditions for the monomer formation give access to a multiplicity of reactive processes, which limits possible control on the reaction outcome [1]. For instance, we have recently demonstrated that the formation of the Cu^(0)^ pre‐nucleation intermediates from Cu^(I)^ precursors in oleylamine can follow competing disproportionation and reduction pathways. The balance between these two processes heavily depends on the nature of the precursor and the reaction conditions [9]. In the same study, we provide evidences that the formation of Cu^(0)^—Cu^(0)^ bond is an essential driving force of the reaction. Having these elements in mind, we turned our attention toward the recent description of a room temperature synthetic process based on the thermal decomposition of an alkyl copper reagent [10]. An organogrignard RMgX reacts in the presence of Cu^(I)^ to form Cu NPs upon addition of dodecanethiol (DDT). Interestingly, the analysis by TEM of the precipitate obtained in ethanol from the reaction mixture before addition of DDT demonstrate that polydisperse spheroid NPs are already present at that stage. Their (moderate) stabilization is proposed to be ensured by the phosphine ligands and the role of DDT is associated to an phosphine / thiol exchange similar or associated to digestive ripening [11]. Initial formation of Cu^(0)^ NPs thus takes place before addition of DDT, and thus upon decomposition of the intermediate alkyl copper. Such formation of Cu^(0)^ precursors through decomposition of main group organometallic reagents is unusual, but not unknown, in the context of Cu NPs synthesis. Indeed, whereas mesitylcopper has been used as a precursor [12, 13], the synthetic processes at stake with this species also include a reducing agent [14, 15, 16]. The formation of alkyl copper through transmetallation of Grignard reagent followed by its decomposition is, however, well‐known in organometallic chemistry. The reaction of ethylmagnesium with copperchloride was first described in 1936, with formation of bulk Cu^(0)^, ethylene and ethane [17]. The formation of an intermediate alkylcopper complex (Equation 1) was first mentioned in 1964 [18] whereas its decomposition mechanism was described a few years later [19, 20]. The process leading to the major organic products is proposed to involve a two‐step mechanism. First, the ẞ‐H elimination step (Equation 2) yields the corresponding alkene and an (unobserved) hydrido‐copper intermediate. Second, a reductive elimination step (Equation 3) yields the corresponding alkane and two Cu^(0)^ centers [20].

(1) $$\begin{array}{ccc} {\text{RCH}}_{2}{\text{CH}}_{2}\text{-MgX} & + & {\text{Cu}}^{\left(I\right)}\text{Cl}\to {\text{RCH}}_{2}{\text{CH}}_{2}{\text{-Cu}}^{\left(I\right)}\\ & & +1/2\;{\text{MgX}}_{2}+1/2\;{\text{MgCl}}_{2} \end{array}$$

(2) $${\text{RCH}}_{2}{\text{CH}}_{2}{\text{-Cu}}^{\left(I\right)}\to \text{RCH}={\text{CH}}_{2}+{\text{Cu}}^{\left(I\right)}H$$

(3) $${\text{Cu}}^{\left(I\right)}H+{\text{RCH}}_{2}{\text{CH}}_{2}{\text{-Cu}}^{\left(I\right)}\to {\text{RCH}}_{2}{\text{CH}}_{3}+2{\text{Cu}}^{\left(0\right)}$$

At the same time, kinetic monitoring of the organic‐by products formation evidenced an autocatalytic process [20], and the model proposed at that time shares common features with those developed in the field of nanoparticle synthesis, where the reduction step takes place in partially reduced clusters (“complex‐aggregative” structures) whose formation facilitates (“auto‐catalyses”) the reduction process [21]. However, it was at that point unclear whether the catalytic behavior should be associated to the ẞ‐H elimination step and thus the formation of intermediate Cu^(I)^ pre‐nucleation species as initially proposed [21] or to the reduction step thus resulting in a “fast autocatalytic surface growth” [22, 23].

To our knowledge, the only computational studies of alkylcopper decomposition at the molecular scale focused on reductive coupling of two alkyl [24] or involving non ẞ‐H eliminable compounds [25]. Some of the steps involved here have been examined on surfaces [26, 27] or model clusters [5], but never in the perspective of new Cu^(0)^ centers formation. None of these preliminary results can be directly applied to the competition examined here and quantitative knowledge of the dynamic equilibria between alkylcopper, hydrido‐copper and Cu^(0)^ including intermediates is still missing. In order to allow such evaluation, we report here a computational study of the individual ẞ‐H elimination and reductive elimination steps in ethylcopper decomposition, in absence of Cu^(0)^ or in presence of one Cu^(0)^ atom, a form proposed to be at stake in the acceleration phase [21]. In a first part, model transition states associated to these reaction steps are described and the corresponding activation energies discussed in order to determine the factors influencing the reaction barriers. In a second step, an overall decomposition scheme is discussed, allowing taking into account the relative stability of all intermediates. Finally, confrontation to experimental data from the literature is carried out, sustaining the reliability of our model.

## 2 Results and Discussion

### 2.1 Model Transition States for Decomposition

Ethylcopper complexes were used as model (R = H in Equations (1), (2), (3)) as decomposition is experimentally reported for EtCu(PR_3_) [28]. A single L = P(CH_3_)_3_ ligand (also noted PMe3) per Cu atom is added to complete the cluster coordination sphere. The starting alkyl copper is thus modelled by Cu(CH_2_CH_3_)L (further referred as CuEtL). Details on the computational level as well as evaluation of the validity of this model can be found in Supporting Information.

#### 2.1.1 Decomposition in Absence of Cu^(0)^

This model was used to evaluate the thermodynamics of Equations 2 and 3, yielding Equations T1 and T2 (Scheme 1). With this model at hand, the ẞ‐H elimination is slightly endergonic (+12.3 kcal mol^−1^). The associated reaction mechanism was examined and the lowest transition state **TS_1_** yields ΔG^#^ = 32.3 kcal mol^−1^ (see Figure 1). From a geometric point of view (see Supporting Information for detailed geometries of intermediates), the transition state exhibits expectedly [29] similar C—H and Cu—H bonds (1.58 and 1.61 Å, respectively) and a slightly shortened C—C bond (1.45 Å with respect to 1.54 Å in the starting material and 1.39 Å in the coordinated ethylene of the connecting product).

**SCHEME 1:**
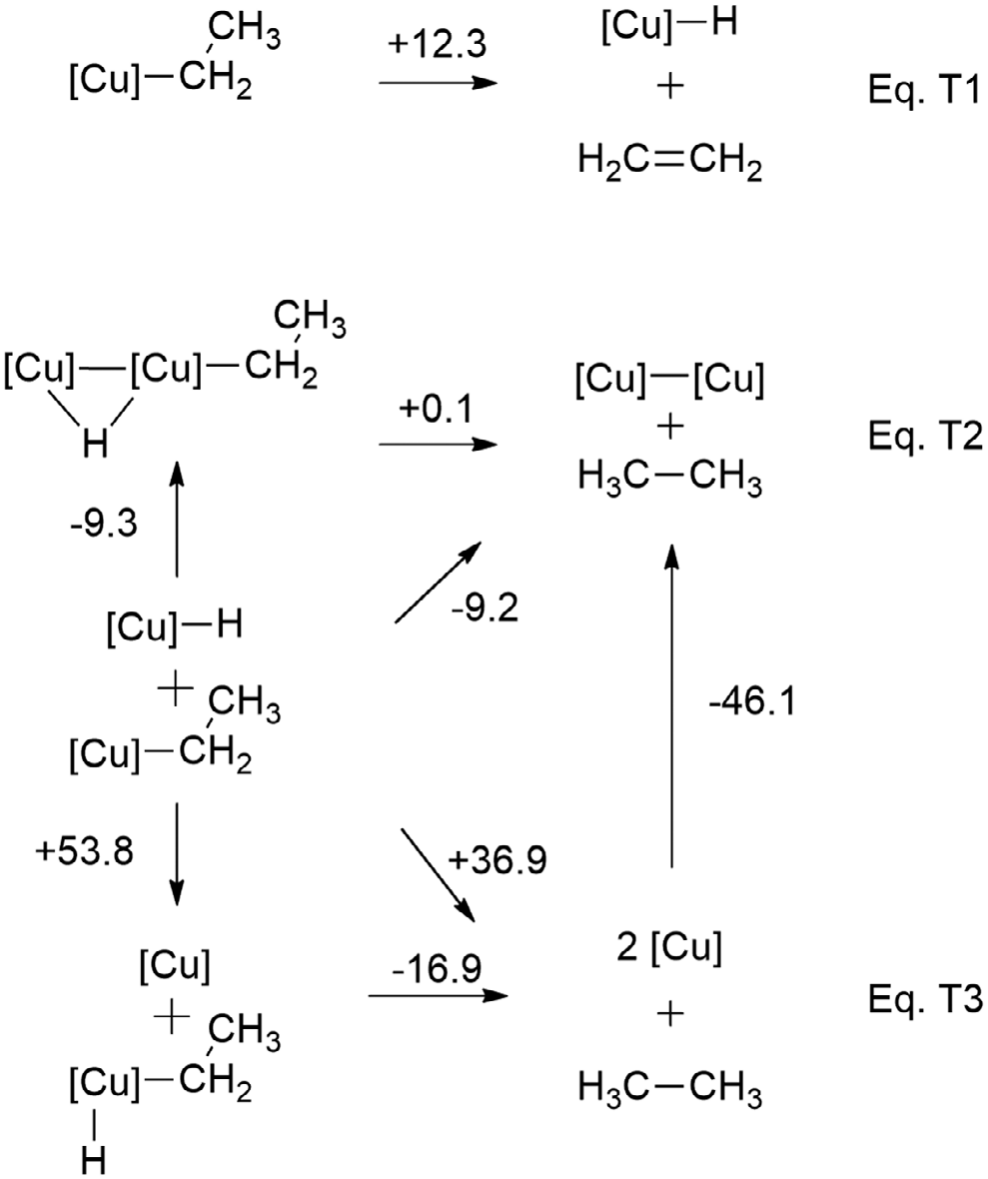
Thermodynamic (Δ_r_G^0^ in kcal mol^−1^) evaluation of individual steps of the reaction. [Cu] stands for Cu and the associated phosphine ligand.

**FIGURE 1:**
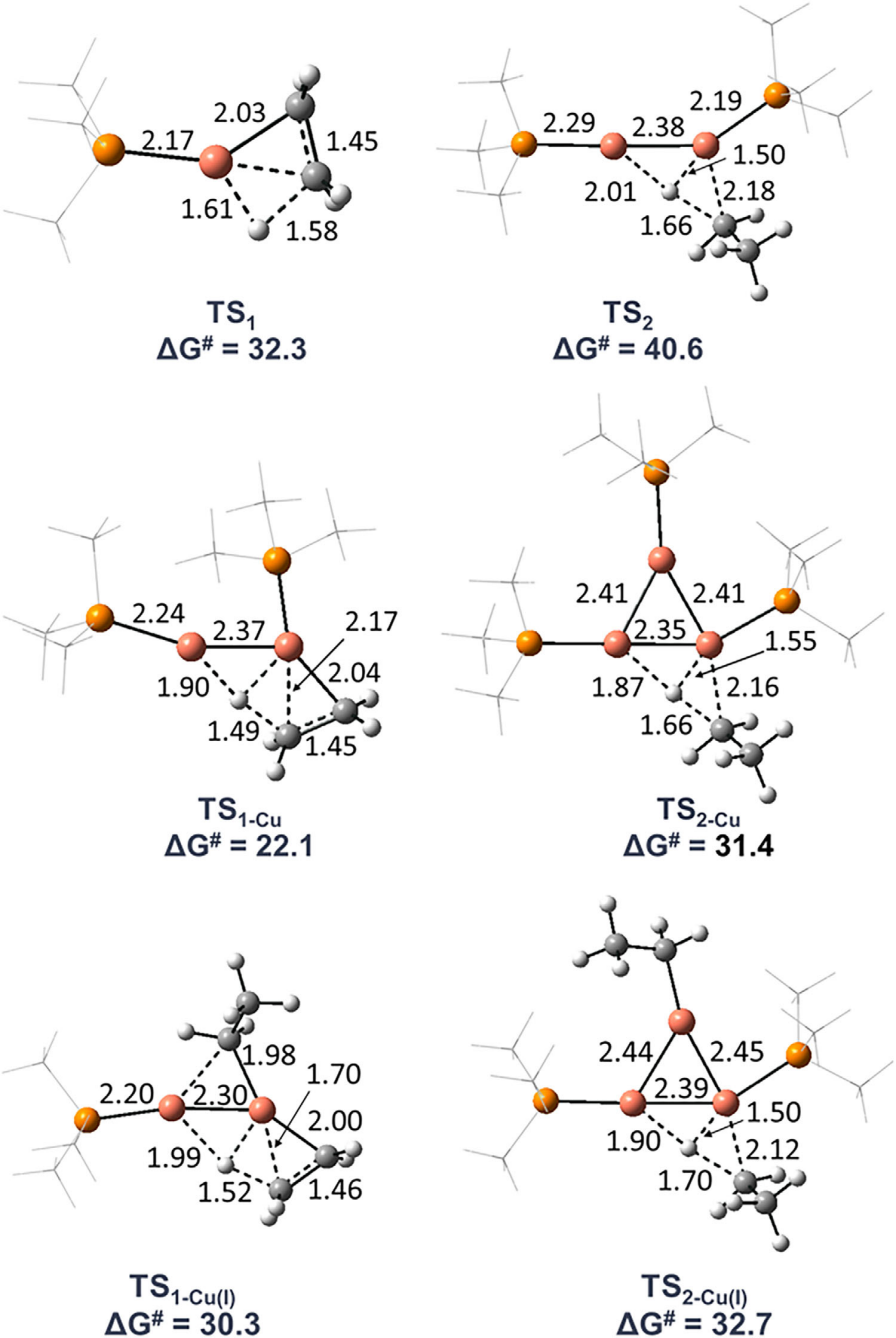
Geometries (distances in Å) and activation barriers (Gibbs energies, in kcal mol^−1^, connecting reactants used as reference) of the transition states for ẞ‐H elimination (eq. T1 top left, eq. T1‐Cu middle left and eq. T1‐Cu^(I)^ bottom left) and reduction (eq. T2 top right, eq. T2‐Cu middle right and T2‐Cu^(I)^ bottom right).

Next, we move on to the reduction step. In order to bring together the reacting hydride and alkyl chains, two hypotheses are tested (see Scheme 1, bottom). In the first one, a disproportionation step is examined, leading to the formation of Cu^(0)^ and a Cu^(II)^ complex (Scheme 1, bottom). This reaction is highly endergonic (+53.8 kcal mol^−1^, a value close to those reported in ref [9]). The subsequent reduction of Cu^(II)^ to Cu^(0)^ is exergonic (eq. T3, −16.9 kcal mol^−1^, see Figure S1 for transition state **TS_3_**). However the overall process leading to formation of two Cu^(0)^ entities is found to be highly endergonic (+36.9 kcal mol^−1^). In the second one, the dimerization of the two Cu^(I)^ centers is proposed. It yields a hydride‐bridged intermediate with a terminal alkyl group and two phosphine ligands. Formation of this intermediate is found to be exergonic by about 10 kcal mol^−1^. In these conditions, a dimeric Cu_2_L_2_ structure is envisioned to model the reaction product [30, 31, 32]. This reaction product is strongly favored considering the high exergonicity of the formation of the Cu^(0)^—Cu^(0)^ bond (46.1 kcal mol^−1^) [9]. With this model, the overall reduction step is given by Equation T2 and is found to be quasi athermic (+0.1 kcal mol^−1^). The reaction pathway for this model reduction step exhibits a high activation barrier is obtained (ΔG^#^ = 40.6 kcal mol^−1^) with a transition state **TS_2_** (Figure 1) where the formation of the C—H bonds takes place in the vicinity of the forming Cu—Cu bond.

#### 2.1.2 Decomposition in Presence of One Spectator Cu Center

Having this model for the non‐assisted reaction pathway at hand, we decided to examine the auto‐catalytic behavior. The seminal work on the decomposition mechanism can be used to propose a model for this behavior. The EPR monitoring of the solution was carried out and an‐EPR active species was observed during the decomposition period. A binuclear mixed Cu^(I)^/Cu^(0)^ species such as RCH_2_CH_2_‐Cu^(I)^Cu^(0)^ is proposed for this reaction intermediate [20]. Such intermediates are reminiscent of the “complex‐aggregative” pathway, where ultra‐small partially reduced clusters are at the heart of the nucleation process [21]. This results in the proposal of Equation T1‐Cu and Equation T2‐Cu in Scheme 2 as alternative models to study the reaction course. They are obtained by coordinating a Cu^(0)^ atom (along with an additional PMe_3_ ligand) in the [Cu] center of Equations T1 and T2. Such coordination (see Supporting Information for detailed geometries) slightly decreases the endergonicity of the ẞ‐H elimination step (from +12.3 to 8.4 kcal mol^−1^) and the reduction step becomes mostly athermic (−0.5 kcal mol^−1^).

**SCHEME 2:**
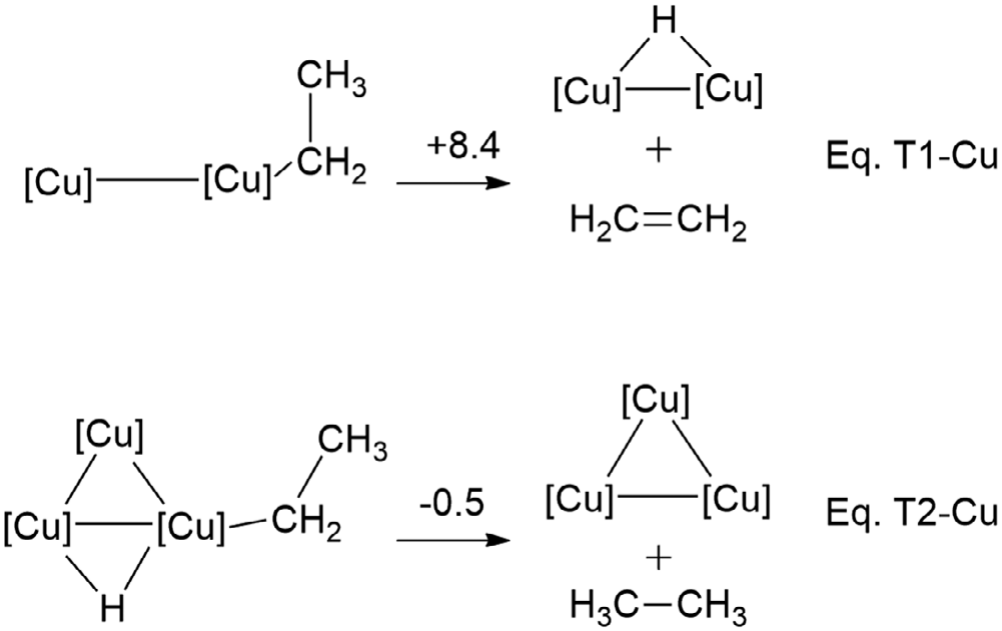
Thermodynamic (Δ_r_G^0^ in kcal mol^−^1) evaluation of individual steps of the reaction using a model including one Cu^(0)^. [Cu] stands for Cu and associated phosphine ligands.

The kinetic effects of this Cu^(0)^ atom are examined by localizing the transition states associated to Equation T1‐Cu and T2‐Cu (Figure 1, center). A significant decrease of the activation barrier is observed, close to 10 kcal mol^−1^ in both ẞ‐H elimination (**TS_1‐Cu_,** 22.1 kcal mol^−1^) and reduction (**TS_2‐Cu_,** 31.4 kcal mol^−1^) steps. However, the geometrical changes for these two steps are very different. In the ẞ‐H elimination process, the addition of an extra Cu‐center enables a geometrical arrangement in **TS_1‐Cu_**, in which the migrating H interacts with two Cu‐centers. This was not possible in **TS_1_**. Minor geometrical changes are observed between **TS_2‐Cu_** and **TS_2_** at the Cu—Cu—H—Et core, with only a slight shortening of the remote Cu–H distance (from 2.01 to 1.87 Å in **TS_2_** and **TS_1‐Cu_**, respectively).

To evaluate the geometrical effects associated to the additional Cu center and the electronic effects associated to its Cu^(0)^ character separately, the same transition states were located using a Cu^(I)^─CH_2_CH_3_ instead of a Cu^(0)^‐PMe_3_ center. This leads to the study of Equation T1‐Cu^(I)^ and Equation T2‐Cu^(I)^ (Scheme 3) and to the optimization of **TS_1‐Cu(I)_** and **TS_2‐Cu(I)_** (Figure 1, bottom). The thermodynamics of the reactions is roughly unaffected (less than 2 kcal mol^−1^ differences) by replacing Cu^(0)^ by Cu^(I)^. In contrast, the kinetic is strongly modified. In the case of the ẞ‐H elimination step, the activation barrier of Equation T1‐Cu^(I)^ (30.3 kcal mol^−1^ for **TS_1‐Cu(I)_**) is closer to that in Equation T1 (32.3 kcal mol^−1^ for **TS_1_**) than in Equation T1‐Cu (22.1 kcal mol^−1^ for **TS_1‐Cu_**). Even though the barrier (32.7 kcal mol^−1^ for **TS_2‐Cu(I)_**) is larger than that for Cu^(0)^ (31.4 kcal mol^−1^), it is still much smaller than in the absence of any Cu center (40.6 kcal mol^−1^ for **TS_2_**). As a conclusion, the presence of the additional Cu center is central for the reduction step, whereas the extra electron associated to the Cu^(0)^ nature seems central for the ẞ‐H elimination.

**SCHEME 3:**
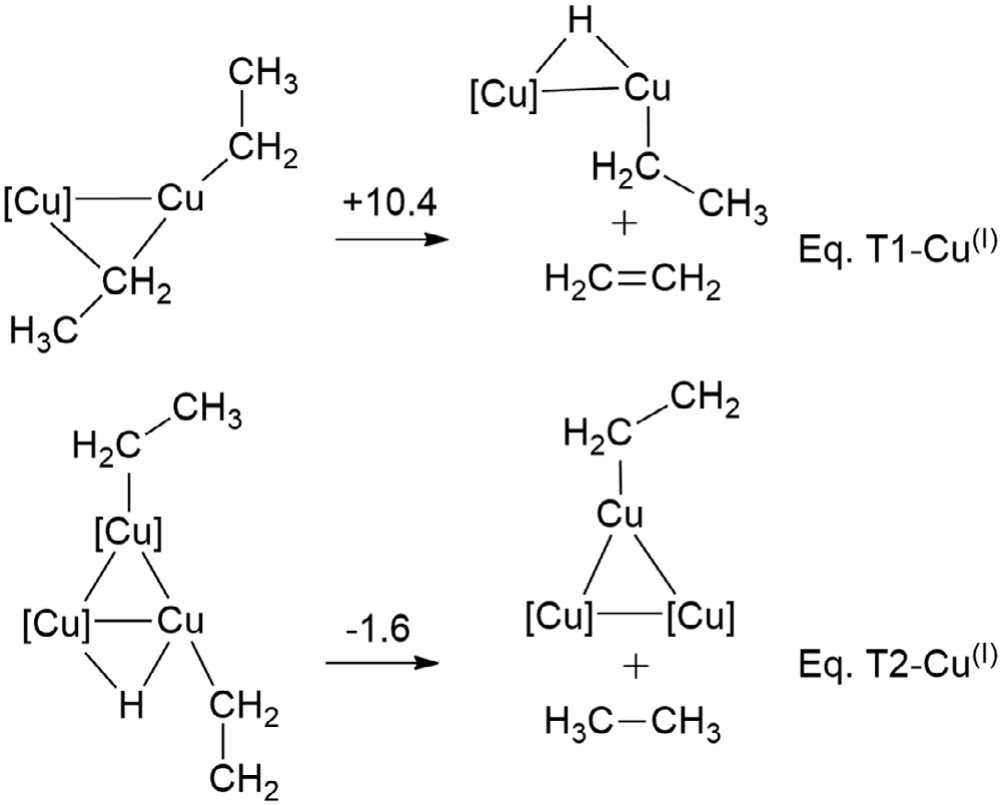
Thermodynamic (Δ_r_G^0^ in kcal mol^−^1) evaluation of individual steps of the reaction using a model including one Cu^(I)^─CH_2_CH_3_. [Cu] stands for Cu and associated phosphine ligands.

Consistently, from a geometrical point of view, the major difference between Cu^(I)^and Cu^(0)^ containing TS is obtained in the case of the ẞ‐H elimination step. The C─H bond breaking is more advanced in the case of Cu^(I)^ (C─H distance equal to 1.52 Å) than in the case of Cu^(0)^ (C─H distance equal to 1.49 Å). It is associated to a longer forming Cu─H bond (1.99 Å for **TS_1‐Cu(I)_** vs. 1.90 Å for TS_1‐Cu(I)_). This highlights a disfavored binding of the forming hydride in **TS_1‐Cu(I)_**, which can be associated to the bridging coordination of the CH_2_─CH_3_ chain in **TS_1‐Cu(I)_** which contrasts with the PMe_3_ ligand in **TS_1‐Cu_**. A similar pattern (longer C─H and Cu─H distances in **TS_2‐Cu(I)_** than in **TS_2‐Cu_**) is observed in the reduction step, however associated to a smaller difference in the binding of the ethyl and phosphine ligands. Notably, the reduction step in **TS_2‐Cu(I)_** is associated with a phosphine ligand migration (from Cu noted a to that noted b in Figure 3) so that this mechanism can be considered as a ligand assisted reduction.

In conclusion, we have demonstrated that the addition of extra Cu centers reduces the activation barrier of the ẞ‐H elimination and reduction steps, with this effect being more pronounced when a Cu^(0)^ center is present, particularly in the case of the ẞ‐H elimination step. However, the models used in this study have been designed to enable comparison with those proposed in the literature [20]. However the relative energies of the proposed intermediates need to be evaluated in order to enable comparison and to examine the overall decomposition mechanism.

### 2.2 Mechanistic Study of the Overall Decomposition

Such an overview of the mechanism requires an adapted model to be determined in order to study the relative stability of the species in solution (Figure 2, see Supporting Information for detailed geometries). As already mentioned, formation of Cu^(0)^ dimers is highly exergonic (Scheme 1) and formation of alkylcopper dimer is kinetically favorable to the reaction. Formation of the Cu_2_L_2_Et_2_ dimer (L = PMe_3_) is found to be exergonic by 3.3 kcal mol^−1^ per monomer, so that it can be considered to be an adapted starting point to model the overall decomposition. Compared to the model used in **TS_1‐Cu(I)_**, this results in adding a second phosphine ligand to the system, whose binding is found to be exergonic by 11.1 kcal mol^−1^. The associated transition state **TS_1‐2P_** was located 27.0 kcal mol^−1^ above the reactant, which shows that addition of this second phosphine slightly decreases the reaction barrier without modifying the conclusion of the previous paragraph. The obtained intermediate **IR_1‐2_**_P_ is an ethylene complex and decoordination of C_2_H_4_ to form the CuL_2_HEt intermediate is found to be quasi athermic. However, since the formation of the intermediate copper hydride dimer **IR_1‐2_**_P_ is endergonic by nearly 10 kcal mol^−1^, **TS_2_** is found to be 50.2 kcal mol^−1^ above the reactants. Using the previously described activated **TS_2‐Cu(I)_** model significantly lowers this value, which is found to be 43.5 kcal mol^−1^ above the Cu_2_L_2_Et_2_ dimer (plus one free phosphine), when the formation of the trimeric form in the Cu‐hydride containing intermediate is taken into account.

**FIGURE 2:**
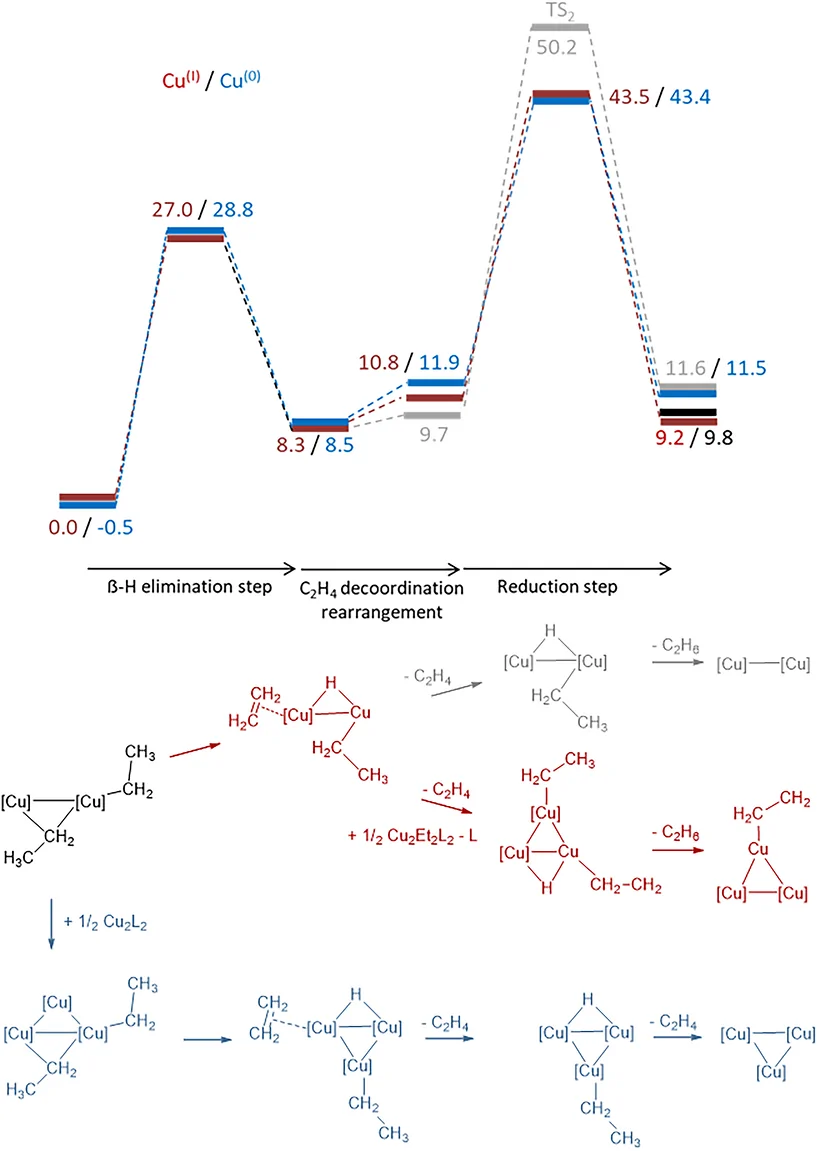
Reaction pathway (Δ_r_G^0^ in kcal mol^−1^ with respect to Cu_2_L_2_Et_2_ dimer, red: most stable pathway, gray: dimeric structure, blue: mixed Cu^(0)^Cu^(I)^ trimeric mechanism). [Cu] stands for Cu and associated phosphine ligands.

The reaction pathway is now being compared in the presence of Cu^(0)^, with the dimeric Cu_2_L_2_ form being considered as the reference state for Cu^(0)^. The formation of a mixed Cu^(0)^Cu^(I)^ dimeric aggregate similar to the one that leads to **TS_1‐Cu_** from Cu^(I)^ and Cu^(0)^ dimers is found to be significantly endergonic (+18.2 kcal mol^−1^). However, a trimeric mixed valence structure similar to those used in **TS_2‐Cu_** was also searched and the mixed valence trimer Cu_3_Et_2_L_3_ is found to be quasi isoenergetic to the reference compounds (Δ_r_G° for Cu_2_Et_2_L_2_ + ½ Cu_2_L_2_ → Cu_3_Et_2_L_3_ equal to 0.5 kcal mol^−1^). Starting from this structure, the new **TS_1mix_** was located for ẞ‐H elimination. A reaction pathway going through **TS_1‐mix_** and then **TS_2‐Cu_** is thus proposed to model the Cu^(0)^ including reaction. The ẞ‐H elimination step is found to exhibit a similar ΔG^#^ for this trimeric mixed valence species than for the dimeric Cu^(I)^ only mechanism. Decoordination of C_2_H_4_ is found to be slightly more endergonic in the case of the mixed Cu^(I)^Cu^(0)^ trimer (+3.4 kcal mol^−1^) than in the case of the Cu^(I)^ only structure so that the stabilization of the reduction step barrier observed previously in presence of a Cu^(0)^ center is canceled by the relative stability of the hydride intermediate. As a consequence, the two trimeric transition states, with Cu^(I)^and with Cu^(0)^, are quasi isoergonic (+43.4 and +43.5 kcal mol^−1^, respectively).

The computational results show that the energies of the Cu^(I)^‐ and Cu^(0)^‐assisted mechanisms are remarkably similar, indicating that both pathways may contribute to the process. Furthermore, the formation of aggregates is found to enhance both steps of the reaction mechanism, thereby improving the overall efficiency of the reaction. However, alternative pathways through dehydrogenation, reductive coupling or hemolytic cleavage can be envisioned for ethylcopper decomposition. Such mechanisms had already been suggested in the experimental studies and are next examined in this perspective.

### 2.3 Confrontation With Experimental Data

Confrontation of these reaction pathways with the experimental data is informative. In one of the first reports on alkylcopper decomposition [19], characterization of the reaction by products starting from n‐butyl‐2,2‐d_2_‐copper was carried out (Scheme 4). In addition to the expected 1‐butene‐d_1_ and butane‐d_3_ products, minor formation of D_2_ and butane‐d_2_ is reported (together with HD).

**SCHEME 4:**
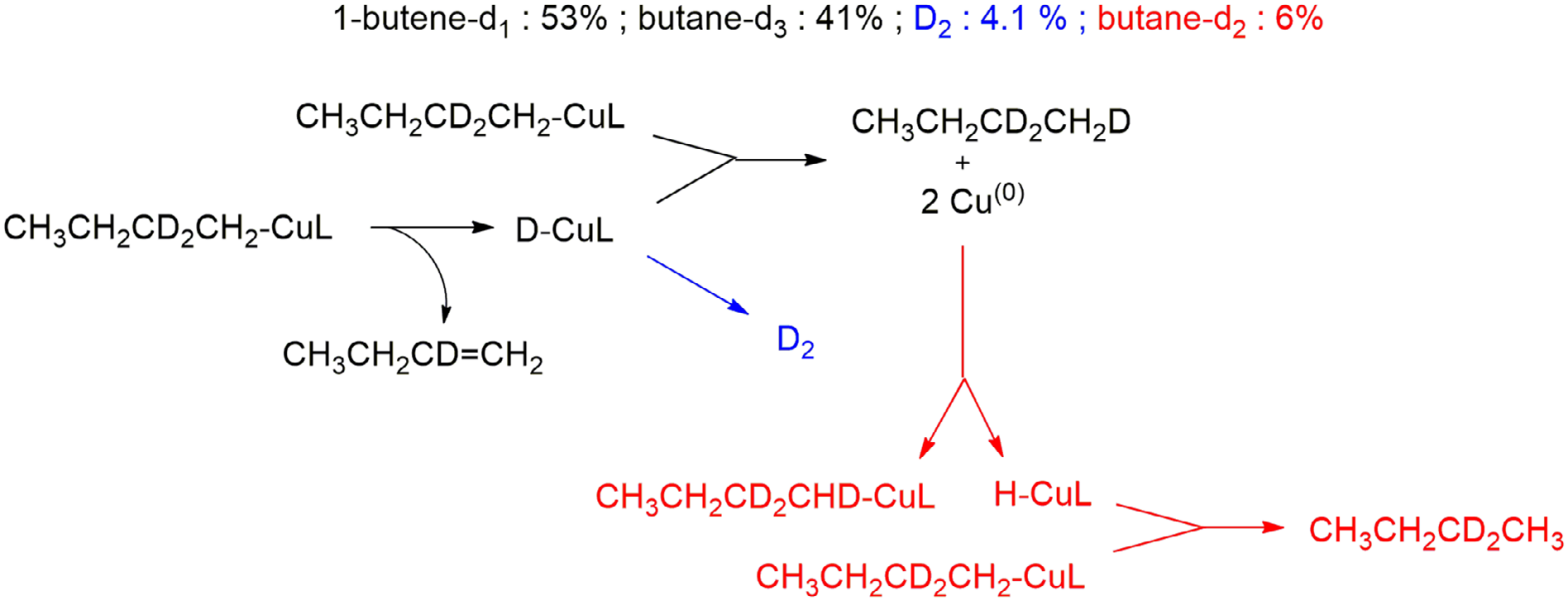
Experimental outcome of the butyl‐copper decomposition (ref 19) with major pathway in black, minor product in blue and proposed pathway for formation of butane‐d^2^ on the basis of computed mechanism in red.

Our finding of an endergonic formation of the hydrido‐copper intermediate is in line with the minimal formation of D_2_. Indeed, a stable hydride intermediate would allow rearrangement of the hydrido‐copper aggregates to form dihydride, subject to dehydrogenation. At the same time, the reductive elimination of ethane is found to be mostly athermic, and thus thermodynamically reversible. This allows us to propose a reversible elimination and possible activation of the terminal C—H bonds of the formed alkane, leading to H/D scrambling in the final species [33]. This reversibility also supports the possibility to resort on Cu NPs for C—H activation catalysis [34].

Experimental kinetic data are also available from thermolysis experiments carried out both in solid state and in solution [28]. First, minor formation of butane is reported upon ethyl‐copper thermolysis (<3%). This reaction product can be proposed to be formed from direct reductive coupling in the dimeric form of Cu^(I)^ reactant Cu_2_Et_2_L_2_. Transition state was obtained (Figure 3) for this bimetallic reductive coupling. The activation barrier is found to be much higher (60.9 kcal mol^−1^) than that obtained in the two‐step reaction mechanism studied in the previous part, in line with the experimental absence or very small formation of the compound. In the same study, the activation energy for the thermolysis of C_2_H_5_Cu(PPh_3_)_2_ was evaluated both in the solid state (48 kcal mol^−1^) and in toluene (32 kcal mol^−1^), whereas a higher value is reported in presence of one single PPh_3_ ligand in the solid state (C_2_H_5_Cu(PPh_3_), E_a_ = 62 kcal mol^−1^). Comparison of these experimental values with computational data can be done using directly SCF energy difference between the reactant and the reduction TS, as the temperature‐dependent linear terms of ΔG^#^ are included in the pre‐exponential factor in an Arhenius‐type evaluation of the activation barrier. We obtain an activation energy of 64.5 kcal mol^−1^ in absence of Cu^(0)^ and 51.8 kcal mol^−1^ in presence of Cu^(0)^ (see Supporting Information for complete thermodynamic data). These values are significantly higher than the value in solution but are in line with that obtained from solid state experiments. Considering our model resort on a single PMe_3_ for the phosphine ligands, the good fit with solid state values can be associated to an insufficient incorporation of solvent—solute interactions.

**FIGURE 3:**
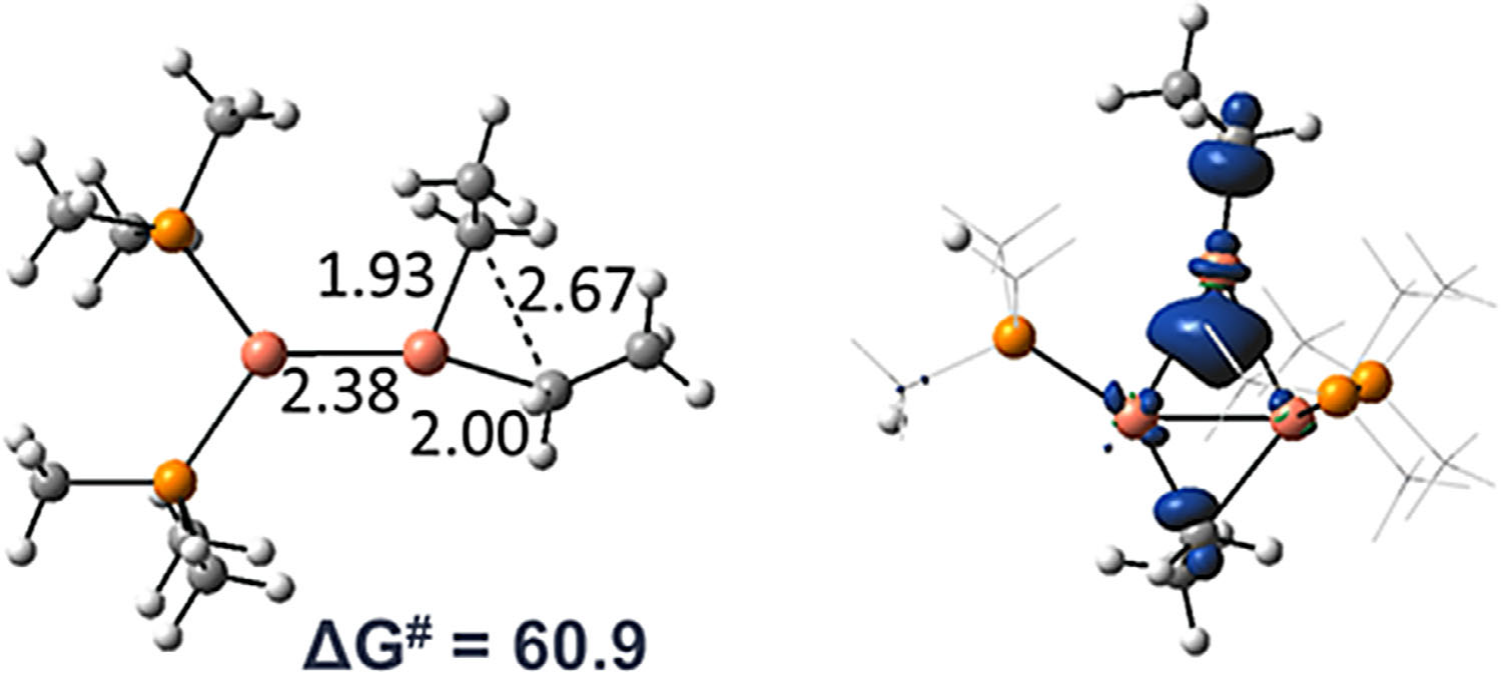
Left: structure (distances in Å) and Gibbs activation energy (in kcal mol^−1^, with respect to connecting reactant) for reductive coupling in Cu_2_Et_2_L_2_; Right: spin density in Cu_3_Et_2_L_3_.

Another experimental study reports EPR monitoring of the reaction mixture revealing the formation, during the decomposition process of paramagnetic species whose multiplicity is in line with “two copper nuclei undergoing spin exchange in (nearly) the same ligand field” (g_iso_ = 2.0024 and A_SU, iso_ = 61 G) [20]. A Cu_2_Et species was proposed by the authors on this basis, which we find too high in energy relative to the reference compounds (18.2 kcal mol^−1^ higher in Gibbs energy that ½ Cu_2_Et_2_L_2_ and ½ Cu_2_L_2_). An alternative mechanism is also proposed with formation of an alkyl radical, which can be also ruled out on the basis of high endergonicity (34.8 kcal mol^−1^ for the reaction of Cu_3_EtL_2_ + L to form Cu_3_L_3_ + Et). However, on the basis of our results, the Cu_3_Et_2_L_3_ aggregate is proposed to be more representative of a stable mixed valence species in presence of Cu^(I)^ and Cu^(0)^ dimers. The corresponding spin density is represented in Figure 3 which evidences localization of the electron density on one a single Cu. In an attempt to check whether a structure bearing an unpaired electron and more than two Cu atoms could yield an EPR signal close to that observed experimentally, the EPR parameters of Cu_3_Et2L3 were evaluated (see computational details) and a g_iso_ of 2.0006117 was obtained (g vector = [1.9921 2.0048 2.0050]). The three Cu centers exhibit very different A_iso_ values as one is very small (−1.81 MHz) compared to the two other ones (116 and 273 MHz, equivalent to 8.3 and 19.5 G). Even though this result cannot be considered as a reliable evaluation, we can safely conclude that the experimental EPR data are consistent with the existence in solution of unsymmetrical mixed valence Cu aggregates with more than two Cu centers.

## 3 Conclusion

We have probed the proposed reaction pathway for decomposition of alkyl copper to Cu^(0)^ aggregates involved in the formation of Cu^(0)^ nanoparticles. A pathway going through ẞ‐H elimination to form ethylene and Cu‐hydride followed by reductive elimination of ethane is shown to be favored over the direct reductive coupling or an alternative pathway involving disproportionation and reductive elimination in a Cu^(II)^ intermediate. Addition of a spectator Cu^(0)^ center on the ẞ‐H elimination or reduction transition states decreases the activation barrier in a more pronounced way than aggregation of an additional Cu^(I)^ center. However, effects of intermediate stabilities and of phosphine binding have to be taken into account to compare the reaction pathways in purely Cu^(I)^ aggregates with that in a mixed Cu^(0)^Cu^(I)^ species. In these conditions, the two reaction mechanisms retained exhibit similar kinetic and thermodynamic properties so that it is not possible to conclude on the basis of our results in an acceleration of the reaction kinetic in presence of Cu^(0)^. In contrast, we can propose that a variety of cluster of different size and oxidation degree can be in equilibrium and active in the reduction mechanism. This hypothesis needs however to be examined in more details.

## Conflicts of Interest

The authors declare no conflicts of interest.

## Supporting Information

The authors have cited additional references within the Supporting Information [35, 36, 37, 38, 39, 40, 41, 42, 43, 44, 45, 46].

## Supporting information




**Supporting File 1**: chem71366‐sup‐0001‐SuppMat.pdf
