# Supplementary material for: Modeling Alkylcopper Decomposition Pathway: Deciphering Pre‐Nucleation Processes for Metallic Cu Nanoparticles Synthesis
Source: Chemistry. 2026 Jul 12;32(39):e71366. doi: 10.1002/chem.71366 (PMC13715448; doi:10.1002/chem.71366)
Supplement: Supplementary file 1 [file CHEM-32-e71366-s001.pdf]

Supplementary information for:

Modelling Alkylcopper Decomposition Pathway: Deciphering Pre-Nucleation Processes for Metallic Cu Nanoparticles Synthesis

Marina Descoubes, Hélène Gérard

Computational details.

Calculations were performed using density functional theory with the Gaussian16 program (rev C.01).[35] Geometry optimisations were carried out without symmetry constraints. The B3PW91 functional[36-37] associated with the 6-31++G(d,p) basis set is used for all atoms except Cu, for which the SDD pseudopotential with associated basis set[38] and f-polarization function[39] is used. This level has been used in continuation to our previous studies were it was shown to proceed efficiently.[9] However, results gathered in Table S1-A showed the importance of adding dispersion effects, which are accounted for using the GD3BJ empirical corrections.[40] Implicit solvation by THF, even though less crucial according to Table S1-A, is modelled using the Self-Consistent Reaction Field method with the default implementation of the Polarizable Continuum Method (PCM).[41] The sensitivity of the results is evaluated *a posteriori* by carrying out single-point computations on the reaction pathway of Figure 2 (see Table S1-B). The most sensitive values are highlighted but don't change the conclusion of the paragraph. For each reaction, various arrangements of the phosphine ligands were tested and only the most stable TS were retained for the overall mechanistic study. The nature of the stationary points was confirmed by harmonic frequency calculations. For transition states (with one and only one imaginary frequency), connectivity with minima was ensured by a small shift along the reaction coordinate followed by geometry optimisation. Gibbs energies are calculated with the standard thermodynamic corrections implemented in Gaussian and corrected using the GoodVibes tool[42] using a 298 K temperature (as reaction can be observed from -63°C et 50 °C), no frequency scaling, a standard state of 1 mol.L^{-1} for all species (so that reported ΔG values can be associated to $\Delta_r G°$ in solution) and default quasi-harmonic entropic corrections with a cut-off of 100 cm^{-1}.[43] Only Gibbs energy values are discussed as many associative and dissociative steps are discussed, which need incorporation of entropic effects at some points. These effects are demonstrated in Table S2. EPR parameters are evaluated using the ORCA software[44] at the same level of theory than geometry optimization. A sample input is given below.

Considering that the reaction can be carried out experimentally with ethyl copper, R = H is used. Phosphine ligands are modelled by PMe_3 adding a single phosphine on each Cu$^{(I)}$ centre. Minor difference in binding energy (see Table S3) is found when comparing to PPh_3 (experimentally, the decomposition was carried out with both triphenyl or trialkyl phosphine). Coordination The small model used is thus expected to properly reproduce electronic effects of the ligand at the metal centre, but is not steric or dispersion effects such as those involved in monolayer formations.[45] As a consequence, coordination / decoordination energies should be taken with caution and are not extensively discussed.

ORCA Input for EPR computation and spin contamination diagnostics.

```
%EPRNMR
    GTENSOR   TRUE
    NUCLEI    = ALL H {AISO, ADIP, AORB}
    NUCLEI    = ALL C {AISO, ADIP, AORB}
    NUCLEI    = ALL P {AISO, ADIP, AORB}
    NUCLEI    = ALL Cu {AISO, ADIP, AORB}
END
----------------------
UHF SPIN CONTAMINATION
----------------------
Expectation value of <S**2>    :    0.755718
Ideal value S*(S+1) for S=0.5  :    0.750000
Deviation                      :    0.005718
```

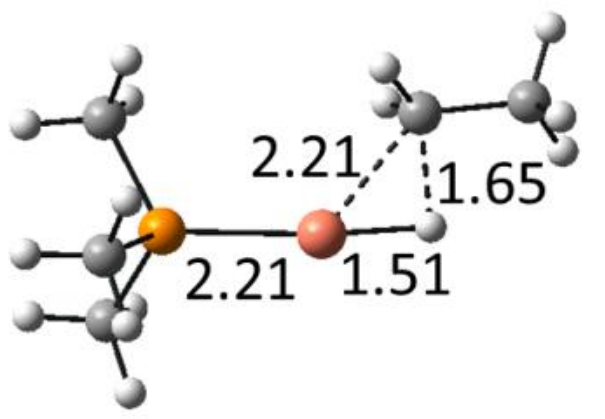

TS_3
$\Delta G^{\#}$ = 14.0

Figure S1: Geometry (distances in Å) and activation barrier (Gibbs energies, in kcal mol^{-1}, taken from the connecting reactant) of the transition states for reductive elimination in Cu(II) disproportionation product.

Table S1-A : comparison of ΔE_{SCF} (in kcal mol^{-1}) for the decomposition of $Cu_2Et_2(PMe_3)_2$ to hydride intermediate and reduced product as a function of the computational level. All structures are reoptimized

B3PW91 / 6-31++G(d,p)/SDD	$Cu_2Et_2(PMe_3)_2$ → $Cu_2HEt(PMe_3)_2$ + C_2H_4	Cu2HEt $(PMe_3)_2$ → Cu_2 $(PMe_3)_2$ + C_2H_6
only	16.4	0.9
+ dispersion (GD3BJ keyword)	25.9	8.0
+ dispersion and PCM (THF)	23.2	8.8

Table S1-B: comparison of ΔE_{SCF} (in kcal mol^{-1}) for the overall decomposition mechanisms (Figure 2) as a function of the computational level (single point computations on the reference level geometries). The most affected features are in bold.

		retained[a]	B3PW91[b]	M06[b]	PBE1PBE[b]	wB97XD[b]
$Cu^{(I)}$	Reactant 1	0.0	0.0	0.0	0.0	0.0
	TS1	30.4	28.3	33.5	28.5	32.3
	Product 1	10.8	8.2	13.3	8.5	10.6
	Reactant 2	28.9	28.5	25.9	30.4	27.6
	TS2	64.5	63.9	62.3	63.8	64.6
	Final products	38.5	39.7	32.0	37.0	31.8
$Cu^{(0)}$	Reactant 1	-6.1	-6.9	-1.6	-3.3	**1.9**
	TS1	26.1	24.1	31.3	27.9	**35.7**
	Product 1	5.5	3.3	9.0	7.6	3.3
	Reactant 2	19.4	17.2	23.2	18.4	21.8
	TS2	51.8	50.6	56.5	51.9	58.1
	Final products	27.6	27.3	28.4	26.6	28.1

a. see computational details; b. The DFT used are referred by the g16 keyword and the Def2TZVP basis set is used.

Table S2: comparison of ΔE_{SCF} and $\Delta_r G°$ (in kcal mol^{-1}) for the overall decomposition mechanisms (Figure 2)

	$Cu^{(I)}$ mechanism		$Cu^{(0)}$ mechanism	
	ΔE_{SCF}	$\Delta_r G°$	ΔE_{SCF}	$\Delta_r G°$
Reactant 1	0.0	0.0	-6.1	-0.5
TS1	30.4	27.0	26.1	28.8
Product 1	10.8	8.3	5.5	8.5
Reactant 2	28.9	10.8	19.4	11.9
TS2	64.5	43.5	51.8	43.4
Final products	38.5	9.1	27.6	11.5

Table S3 : comparison L ligand binding energies (ΔE_{SCF} inkcal mol^{-1}) for various copper complexes

	$Cu^{(0)}$	CuH	CuEt
THF	-7.7	-25.1	-22.4
PH_3	-11.8	-28.5	-26.0
PMe_3	-20.9	-35.0	-36.0
PPh_3	-21.9	-37.8	-35.7

Thermodynamic data and Standard state and quasi-harmonic corrections (qh) (values in Hartree):

Structure	E	ZPE	H	T.S	T.qh-S	G(T)	qh-G(T)
CH2CH2	-78.569593	0.051011	-78.51459	0.023162	0.023162	-78.537752	-78.537752
CH3CH3	-79.81588	0.074612	-79.736837	0.024527	0.024529	-79.761364	-79.761366
CH2CH3	-79.143752	0.059315	-79.079525	0.026055	0.025978	-79.10558	-79.105503
Cu2_Et2_1PMe3	-1014.30697	0.243081	-1014.04443	0.064903	0.061233	-1014.10933	-1014.10566
Cu2_Et2_2PMe3	-1475.38701	0.357755	-1475.00194	0.0818	0.076815	-1475.08374	-1475.07876
Cu2_EtH_2PMe3	-1396.78037	0.301203	-1396.4551	0.075407	0.070891	-1396.53051	-1396.526
Cu2_HEt_PMe3	-935.699144	0.186705	-935.496393	0.05847	0.054935	-935.554863	-935.551328
Cu3_3PMe3	-1975.4328	0.345094	-1975.05714	0.094359	0.08628	-1975.15149	-1975.14342
Cu3_Et2_3PMe3	-2133.87199	0.471943	-2133.36191	0.108413	0.099671	-2133.47032	-2133.46158
Cu3_Et2H_2PMe3	-1673.42246	0.366678	-1673.02607	0.087501	0.081976	-1673.11357	-1673.10804
Cu3_Et_2PMe3	-1593.59177	0.294045	-1593.27128	0.085687	0.077808	-1593.35697	-1593.34909
Cu3_EtH_PMe3	-1594.17931	0.301182	-1593.85176	0.082639	0.076861	-1593.9344	-1593.92862
Cu3_PMe3	-1514.36483	0.230922	-1514.11187	0.073666	0.068518	-1514.18554	-1514.18039
CuCu_2PMe3	-1316.95048	0.229871	-1316.70055	0.068109	0.063508	-1316.76866	-1316.76406
CuCu_Et_2PMe2	-1396.138	0.293302	-1395.82056	0.07761	0.071863	-1395.89817	-1395.89243
CuCu_Et_PMe3	-935.055346	0.178546	-934.860671	0.060923	0.056399	-934.921594	-934.91707
CuCu_H_2PMe2	-1317.53295	0.23605	-1317.27641	0.069449	0.064842	-1317.34586	-1317.34125
CuCu_H_PMe3	-856.453127	0.122085	-856.318917	0.049505	0.047441	-856.368421	-856.366357
Cu_Et_PMe3	-737.677623	0.178381	-737.485742	0.050274	0.048341	-737.536017	-737.534083
Cu_HEt_1P	-738.22709	0.1835	-738.029152	0.052311	0.050548	-738.081463	-738.0797
Cu_H_PMe3	-659.067118	0.120859	-658.936278	0.040549	0.040395	-658.976826	-658.976673
CuPMe3_Cu_Et_PMe3	-1396.13095	0.293071	-1395.81373	0.077075	0.071908	-1395.8908	-1395.88564
CuPMe3_CuPMe3	-1316.94648	0.229489	-1316.69669	0.069531	0.064484	-1316.76622	-1316.76117
Cu_PMe3	-658.428429	0.114308	-658.304452	0.041216	0.040809	-658.345668	-658.345261
IR1_mix	-2133.85349	0.467818	-2133.34718	0.108531	0.100059	-2133.45571	-2133.44724
PMe3	-461.041956	0.112871	-460.921468	0.033867	0.033876	-460.955335	-460.955343
prod_TS1_2PMe3	-1475.3698	0.354032	-1474.98848	0.082312	0.077009	-1475.07079	-1475.06549
prod_TS1CuI	-1014.288	0.239418	-1014.02924	0.065263	0.061295	-1014.0945	-1014.09053
prod_TS1Cu	-1396.13065	0.288841	-1395.81802	0.07531	0.070571	-1395.89333	-1395.88859
prod_TS1	-737.652576	0.174098	-737.4651	0.048948	0.047599	-737.514048	-737.512699
reac_but	-1475.38617	0.357341	-1475.00119	0.083162	0.077607	-1475.08436	-1475.0788
reac_TS1_mix	-2133.87204	0.472029	-2133.36196	0.107526	0.099298	-2133.46948	-2133.46126
reac_TS2CuI	-1673.42296	0.366923	-1673.02648	0.086382	0.081368	-1673.11286	-1673.10785
reac_TS2Cu	-2055.26168	0.416345	-2054.811	0.101577	0.093003	-2054.91257	-2054.904
reac_TS2	-1396.78009	0.301145	-1396.45483	0.075205	0.070793	-1396.53003	-1396.52562
reac_TS3	-738.226673	0.183414	-738.028812	0.052421	0.050644	-738.081233	-738.079456
TS1_2PMe3	-1475.33851	0.352374	-1474.9591	0.082125	0.076618	-1475.04123	-1475.03572
TS1Cu2P	-1396.08886	0.287344	-1395.7778	0.077737	0.071654	-1395.85554	-1395.84945
TS1CuI	-1014.25369	0.237859	-1013.99689	0.064331	0.06054	-1014.06122	-1014.05743
TS1Cu	-1396.09178	0.287937	-1395.78047	0.074203	0.06987	-1395.85467	-1395.85034
TS1	-737.622085	0.173344	-737.435841	0.048263	0.046701	-737.484103	-737.482542
TS1_mix	-2133.8206	0.466787	-2133.31604	0.107208	0.098807	-2133.42325	-2133.41485
TS2CuI	-1673.36615	0.364462	-1672.97142	0.091719	0.084347	-1673.06314	-1673.05576
TS2Cu	-2055.21009	0.41462	-2054.761	0.100673	0.092921	-2054.86167	-2054.85392
TS2	-1396.71229	0.299443	-1396.38839	0.07866	0.07259	-1396.46705	-1396.46098
TS3	-738.20377	0.183131	-738.006361	0.053504	0.050836	-738.059865	-738.057197
TS_but	-1475.28813	0.356919	-1474.90379	0.084417	0.077957	-1474.98821	-1474.98175

Cartesian coordinates and thermodynamic data for the optimized geometries :

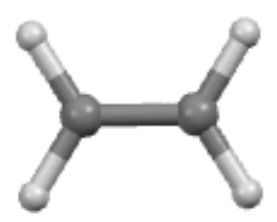

6
System = CH2CH2; C2H4 ; E(SCF) = -78.5695925611 Hartree; G(298.150 K) = -78.540771 Hartree; Convergence on forces and displacements = 4 YES; Minimal frequency = 825.7926 cm-1; S**2 = None (restricted formalism)

C	0.446890	0.175954	0.075796
H	0.024887	0.073055	1.072768
H	0.884861	-0.717246	-0.363526
C	0.428351	1.340007	-0.575794
H	0.850355	1.442908	-1.572766
H	-0.009619	2.233208	-0.136473

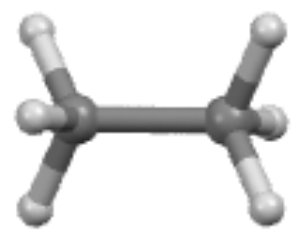

8
System = CH3CH3; C2H6 ; E(SCF) = -79.8158803497 Hartree; G(298.150 K) = -79.764383 Hartree; Convergence on forces and displacements = 4 YES; Minimal frequency = 309.4545 cm-1; S**2 = None (restricted formalism)

C	0.107658	0.106757	0.067277
H	0.376923	0.096742	1.129045
H	0.477891	-0.824039	-0.376008
C	0.687150	1.328380	-0.641230
H	0.417882	1.338397	-1.702998
H	0.316920	2.259175	-0.197943
H	-0.985807	0.090558	0.003803
H	1.780615	1.344576	-0.577760

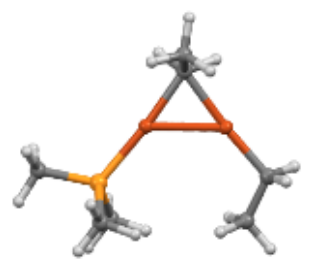

29
System = Cu2_Et2_1PMe3; C7H19Cu2P ; E(SCF) = -1014.30696622 Hartree; G(298.150 K) = -1014.112349 Hartree; Convergence on forces and displacements = 3 YES; Minimal frequency = 28.8489 cm-1; S**2 = None (restricted formalism)

Cu	3.100185	3.434027	0.535481
C	4.876751	3.745689	1.428660
Cu	4.680798	1.709971	0.951427
C	3.448274	-0.630326	-0.257307
C	4.619654	-0.188500	0.635094
H	2.480819	-0.334275	0.168185
H	3.504329	-0.169432	-1.251705
H	3.406175	-1.720972	-0.415212
H	5.566969	-0.526005	0.183380
H	4.556146	-0.712333	1.603393
C	4.714051	4.057085	2.926194
H	4.707294	3.140163	3.526008
H	5.890670	3.330199	1.247920
H	4.927617	4.688369	0.852132
H	5.522075	4.694534	3.311561
P	1.105897	3.280995	-0.373958
C	1.117792	2.324305	-1.935214
H	1.741549	2.836482	-2.672213
H	0.103610	2.213042	-2.331080
C	-0.094379	2.376329	0.673120

H	0.307739	1.387778	0.908543
H	-1.053782	2.265857	0.158138
H	-0.245912	2.920659	1.608742
H	0.072742	5.427441	0.086029
C	0.221824	4.824354	-0.813262
H	0.823762	5.399391	-1.521501
H	-0.750093	4.599454	-1.263423
H	3.768806	4.572905	3.131029
H	1.545885	1.337148	-1.744995

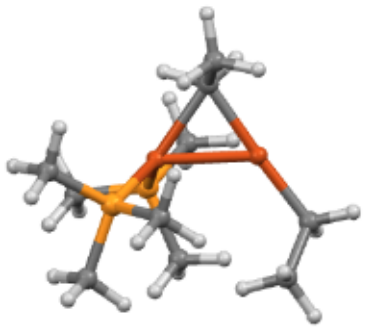

42

System = Cu2_Et2_2PMe3; C10H28Cu2P2 ; E(SCF) = -1475.38700836 Hartree; G(298.150 K) = -1475.086762 Hartree; Convergence on forces and displacements = 4 YES; Minimal frequency = 15.4012 cm-1; S**2 = None (restricted formalism)

C	0.893966	4.035178	2.524001
P	0.824929	2.362496	1.767253
C	2.346003	2.322863	0.738036
C	-0.489991	2.545115	0.500337
Cu	0.566662	0.592595	3.093598
C	-1.398127	0.231795	3.934938
C	-2.574444	1.175302	3.646245
P	2.218301	-0.814194	3.632499
C	3.774752	-0.199412	4.395539
Cu	-1.058475	-0.944185	2.314405
C	-0.833585	-2.096564	0.776648
C	-0.168064	-1.403989	-0.424601
C	1.753036	-2.201689	4.740748
C	2.827044	-1.693926	2.141595
H	0.790546	-0.945693	-0.148714
H	-1.797855	-2.525906	0.455272
H	-0.221347	-2.965676	1.075017
H	0.034372	-2.080937	-1.272081
H	-0.795173	-0.592583	-0.815697
H	-0.718786	0.741268	4.658776
H	-1.746491	-0.630966	4.529874
H	-3.118563	1.481816	4.552906
H	-2.232906	2.090658	3.150184
H	-3.307285	0.710902	2.974429
H	1.013030	4.811415	1.760904
H	-0.028853	4.216832	3.081585
H	1.733697	4.084201	3.222560
H	2.398918	3.185019	0.064834
H	3.224692	2.316618	1.388610
H	2.349668	1.403948	0.145441
H	-0.283028	3.380988	-0.176078
H	-0.555778	1.616976	-0.072585
H	-1.450440	2.705772	0.995878
H	2.578618	-2.909687	4.868301
H	1.460164	-1.806432	5.717264
H	0.892047	-2.717856	4.307167
H	3.585180	-2.439803	2.401890
H	1.979924	-2.183812	1.654922
H	3.255390	-0.970740	1.442757
H	4.495414	-1.010639	4.544116
H	4.217167	0.561018	3.746192
H	3.549668	0.261966	5.361131

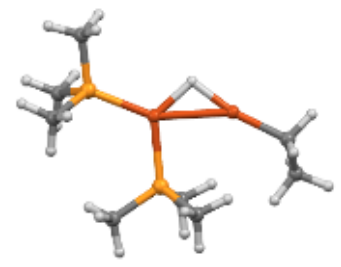

36

System = Cu2_EtH_2PMe3; C8H24Cu2P2 ; E(SCF) = -1396.78037372 Hartree; G(298.150 K) = -1396.533529 Hartree; Convergence on forces and displacements = 3 YES; Minimal frequency = 11.3372 cm-1; S**2 = None (restricted formalism)

C	2.079410	1.152082	-1.855315
C	2.600517	2.365298	-1.068613
Cu	0.928848	-0.104214	-0.920541
Cu	-1.119671	-0.600718	0.429994
H	0.136586	-1.366832	-0.285198
H	2.943967	0.586040	-2.243241
H	1.552430	1.510911	-2.756809
H	3.147904	2.061049	-0.166874
H	3.283564	3.005716	-1.651524
H	1.782879	3.014338	-0.729526
P	-1.356975	1.627697	0.700662
C	-2.871307	2.238388	1.547769
C	-0.018448	2.347942	1.727956
C	-1.324651	2.651335	-0.820659
H	-1.376739	3.718268	-0.580341
H	-0.402980	2.435340	-1.366952
H	-2.173849	2.381246	-1.454535
H	-0.135161	3.432052	1.827487
H	-0.039128	1.889699	2.720591
H	0.944072	2.124840	1.262037
H	-2.847298	3.326070	1.673088
H	-3.750864	1.967087	0.957557
H	-2.955287	1.766365	2.530459
P	-2.655465	-1.927951	1.405078
C	-4.411615	-1.471458	1.118607
C	-2.642659	-3.720916	1.011369
C	-2.539556	-1.920390	3.238417
H	-5.089130	-2.150145	1.647088
H	-4.580047	-0.450230	1.469219
H	-4.628528	-1.508979	0.047639
H	-3.305672	-2.562552	3.684966
H	-1.550974	-2.275265	3.541699
H	-2.667008	-0.898577	3.605254
H	-3.414165	-4.259096	1.571917
H	-2.813636	-3.856107	-0.059998
H	-1.660823	-4.134864	1.255766

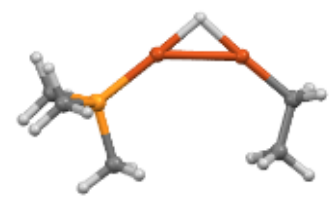

23

System = Cu2_HEt_PMe3; C5H15Cu2P ; E(SCF) = -935.699143795 Hartree; G(298.150 K) = -935.557882 Hartree; Convergence on forces and displacements = 4 YES; Minimal frequency = 22.0915 cm-1; S**2 = None (restricted formalism)

Cu	2.109307	2.437767	0.351992
Cu	4.297078	3.117935	1.152659
H	3.155426	2.084175	1.558520
C	0.819631	2.795826	-1.039536
H	-0.152560	3.026279	-0.573458
H	0.653616	1.873148	-1.619669
C	1.208381	3.935292	-1.995730
H	1.347981	4.883409	-1.461282
H	2.154958	3.727440	-2.510377
H	0.454577	4.120044	-2.779014
P	5.914267	4.525370	0.640427
C	6.360353	5.736900	1.938433

C	7.513226	3.759818	0.183641
C	5.493924	5.563004	-0.808106
H	7.362695	3.079296	-0.658118
H	8.241181	4.527430	-0.096478
H	7.898784	3.186506	1.030401
H	6.720808	5.206647	2.823494
H	7.140279	6.415943	1.580127
H	5.475661	6.315612	2.215446
H	6.313219	6.248867	-1.044532
H	5.297987	4.919663	-1.669575
H	4.589190	6.136937	-0.592932

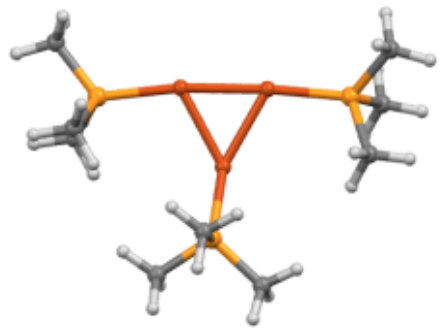

42

System = Cu3_3PMe3; C9H27Cu3P3(2) ; E(SCF) = -1975.43279907 Hartree; G(298.150 K) = -1975.154513 Hartree; Convergence on forces and displacements = 3 YES; Minimal frequency = 10.5524 cm-1; S**2 = 0.7595

C	-0.975635	-2.345373	4.609954
P	-0.640593	-1.179641	3.229928
C	-2.068839	-1.469981	2.109595
C	0.740812	-1.999361	2.337791
Cu	-0.270017	0.972215	3.775480
Cu	-0.202167	2.981431	2.461763
P	1.088445	2.755602	0.637502
C	0.348248	1.553388	-0.540305
Cu	-1.458648	2.952404	4.423648
P	-2.605131	2.773243	6.343623
C	-3.249665	1.061592	6.539390
C	-4.106221	3.790187	6.662387
C	-1.645539	3.009430	7.894535
C	1.472887	4.188195	-0.450242
C	2.753853	2.028992	0.927491
H	-4.576076	3.523787	7.615568
H	-3.828775	4.847745	6.679828
H	-4.822358	3.637928	5.850315
H	-2.257266	2.795057	8.777635
H	-0.778204	2.344281	7.880608
H	-1.286993	4.041159	7.946579
H	-3.793994	0.944820	7.482634
H	-3.916619	0.827547	5.705184
H	-2.408606	0.363811	6.513716
H	-2.172990	-2.531330	1.859198
H	-2.982899	-1.120494	2.596898
H	-1.930767	-0.892178	1.192185
H	0.488884	-3.033056	2.078802
H	0.963332	-1.442916	1.423394
H	1.633587	-1.991344	2.968844
H	-1.130821	-3.364499	4.240479
H	-0.131565	-2.336869	5.304782
H	-1.868768	-2.018063	5.148980
H	1.005414	1.381964	-1.399373
H	0.175367	0.606163	-0.023219
H	-0.615434	1.932711	-0.890040
H	3.285100	1.848895	-0.013839
H	3.340916	2.708746	1.550968
H	2.636542	1.086909	1.469666
H	2.073504	3.883045	-1.313912
H	0.538906	4.637038	-0.798703
H	2.022505	4.940085	0.122759

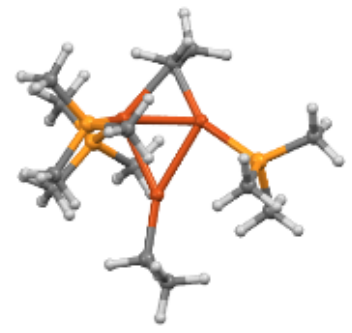

56
System = Cu3_Et2_3PMe3; C13H37Cu3P3(2) ; E(SCF) = -2133.87198849 Hartree; G(298.150 K) = -2133.473337 Hartree; Convergence on forces and displacements = 2 YES; Minimal frequency = 15.1629 cm-1; S**2 = 0.7555

Cu	1.159864	1.130676	1.875630
Cu	-0.791279	0.121474	0.555743
Cu	1.627576	-0.343648	0.087053
C	-2.651136	-0.231419	0.046284
H	-3.093184	-0.895462	0.806778
H	-2.624936	-0.813667	-0.889220
C	-3.525553	1.012147	-0.137887
H	-3.108844	1.692116	-0.892587
H	-4.556698	0.781366	-0.456150
H	-3.607986	1.590077	0.791463
C	3.068238	0.512038	1.616039
H	3.533661	1.509830	1.641650
H	3.516877	0.054022	0.706452
C	3.504105	-0.315456	2.830875
H	3.075080	-1.322898	2.796314
H	3.167301	0.135543	3.772330
H	4.597344	-0.433225	2.902851
P	-0.358865	2.410206	2.846163
C	0.086338	3.574129	4.207834
C	-1.175457	3.532067	1.644119
C	-1.774603	1.498917	3.577664
H	0.494529	3.006552	5.048684
H	0.855641	4.266138	3.854083
H	-0.784829	4.145409	4.547815
H	-0.432210	4.208687	1.213826
H	-1.963963	4.118763	2.126687
H	-1.606816	2.928806	0.841621
H	-2.526118	2.191425	3.970931
H	-2.216941	0.866871	2.802280
H	-1.414665	0.856375	4.385983
P	1.275075	-2.530056	0.311657
C	2.656203	-3.748962	0.217799
C	0.091037	-3.200553	-0.922803
C	0.471860	-3.006285	1.895393
H	-0.816655	-2.591495	-0.897890
H	-0.159513	-4.245853	-0.713100
H	0.529574	-3.127680	-1.921653
H	3.390134	-3.521491	0.996096
H	2.298566	-4.776318	0.349481
H	3.149832	-3.660795	-0.754073
H	1.120831	-2.728678	2.730240
H	-0.460897	-2.442781	1.988188
H	0.264050	-4.081156	1.935726
P	1.471022	0.758320	-1.846490
C	2.895267	0.880516	-3.014527
C	0.131756	0.153951	-2.950094
C	1.016954	2.527950	-1.642114
H	0.012279	0.800974	-3.825716
H	0.363591	-0.861967	-3.280578
H	-0.800325	0.125344	-2.378884
H	0.917715	3.036230	-2.607398
H	0.070117	2.576153	-1.098118
H	1.780804	3.029054	-1.041103

H	2.642341	1.471252	-3.902228
H	3.740910	1.347649	-2.501605
H	3.195741	-0.124395	-3.324239

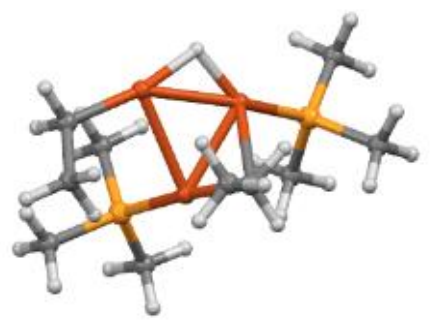

44

System = Cu3_Et2H_2PMe3; C10H29Cu3P2 ; E(SCF) = -1673.42246358 Hartree; G(298.150 K) = -1673.116586 Hartree; Convergence on forces and displacements = 2 YES; Minimal frequency = 26.7429 cm-1; S**2 = None (restricted formalism)

C	-1.696398	-1.808512	2.275138
P	-0.255507	-0.824249	2.833364
C	-1.034064	0.382736	3.977592
Cu	1.101531	0.029287	1.298297
Cu	3.456838	-0.120638	1.864264
P	3.926809	1.487731	3.319906
C	2.504869	1.985056	4.367202
Cu	2.521894	-2.187715	0.907405
C	1.027864	-3.207533	0.207336
C	0.082977	-2.465826	-0.746979
C	0.628531	-1.962450	3.963814
C	4.393024	3.071160	2.517452
C	5.265115	1.239851	4.551014
H	3.931121	-1.628210	1.500324
H	0.455047	-3.624768	1.051754
H	1.440975	-4.088700	-0.312522
H	0.599702	-2.173975	-1.667944
H	-0.295254	-1.531416	-0.305994
H	-0.799802	-3.056099	-1.044360
H	2.760843	2.838591	5.003096
H	1.666707	2.252201	3.718520
H	2.206078	1.145281	4.999565
H	4.562408	3.856836	3.260674
H	5.303113	2.927932	1.928969
H	3.590534	3.376646	1.840944
H	5.381855	2.119852	5.192130
H	5.029691	0.369650	5.169551
H	6.205372	1.045445	4.028441
H	-0.038155	-2.343610	4.743754
H	1.029025	-2.791793	3.375470
H	1.468922	-1.435622	4.422839
H	-1.643006	-0.131314	4.728106
H	-0.262504	0.967452	4.482599
H	-1.668107	1.066454	3.406901
H	-2.256808	-2.187591	3.135546
H	-2.351066	-1.180119	1.665816
H	-1.351088	-2.645257	1.665642
C	2.273657	1.026145	0.064225
H	3.351532	1.191154	0.260756
C	2.136526	0.473961	-1.359885
H	1.849577	2.041759	0.125968
H	2.650832	1.098152	-2.106447
H	2.557249	-0.536133	-1.428533
H	1.087560	0.398319	-1.666981

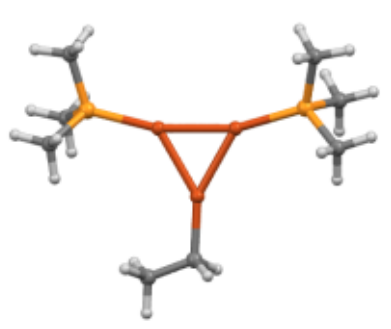

36

System = Cu3_Et_2PMe3; C8H23Cu3P2 ; E(SCF) = -1593.59176940 Hartree; G(298.150 K) = -1593.359986 Hartree; Convergence on forces and displacements = 4 YES; Minimal frequency = 11.1330 cm-1; S**2 = None (restricted formalism)

C	0.187481	-0.317810	0.705415
P	1.651240	0.072101	1.742065
C	3.010921	-0.819050	0.890805
Cu	1.537289	-0.352900	3.951951
Cu	3.155278	-0.339663	5.712575
C	5.015990	-0.063573	6.226821
C	5.930435	0.344088	5.062116
Cu	0.853866	-0.878949	6.160098
P	-0.363942	-1.443085	7.976549
C	-1.896179	-2.412652	7.689977
C	1.994089	1.827322	1.330625
C	0.551346	-2.469037	9.192194
C	-0.953547	-0.032776	8.992531
H	-0.046467	-1.382086	0.793545
H	-0.672613	0.255767	1.061026
H	0.374426	-0.074694	-0.345657
H	1.181551	2.454785	1.706215
H	2.922279	2.136395	1.818425
H	2.089051	1.966193	0.248804
H	2.850750	-1.896762	0.979705
H	3.058677	-0.545185	-0.168207
H	3.960226	-0.571022	1.372756
H	1.445998	-1.931234	9.516671
H	0.864608	-3.401983	8.716258
H	-0.071529	-2.697267	10.063237
H	-1.499553	-0.383989	9.874195
H	-1.609669	0.600485	8.389525
H	-0.096047	0.564894	9.312615
H	-1.645425	-3.342818	7.173144
H	-2.576488	-1.837725	7.056064
H	-2.395232	-2.647834	8.635722
H	5.396791	-0.988535	6.690844
H	5.057077	0.704886	7.016584
H	5.598232	1.280196	4.595073
H	6.981563	0.495831	5.362496
H	5.936375	-0.413407	4.267826

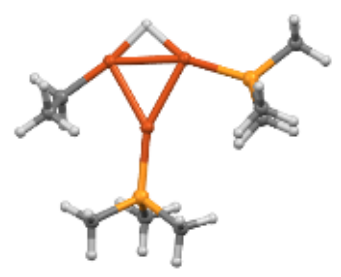

37

System = Cu3_EtH_PMe3; C8H24Cu3P2(2) ; E(SCF) = -1594.17930843 Hartree; G(298.150 K) = -1593.937416 Hartree; Convergence on forces and displacements = 4 YES; Minimal frequency = 15.5773 cm-1; S**2 = 0.7552

C	1.809331	-1.343612	-1.239286
C	1.004931	-0.146457	-1.761744
Cu	3.534327	-0.880409	-0.461876
Cu	2.155114	-0.071563	1.385153
P	0.136828	-0.247620	2.319266
Cu	4.645968	0.124683	1.397809
P	5.010877	0.979907	3.400013
H	5.102558	-0.594804	-0.018495
H	1.194572	-1.924573	-0.534012
H	2.030803	-2.034940	-2.070419
H	1.567257	0.419916	-2.514023
H	0.769466	0.562050	-0.956024
H	0.046844	-0.434480	-2.224239
C	-1.317360	-0.237860	1.199790
C	-0.085950	-1.802998	3.268041

C	-0.279850	1.046710	3.551999
C	6.676845	1.355809	4.089358
C	4.275675	-0.091475	4.697371
C	4.120834	2.567686	3.636065
H	4.198199	2.911189	4.672730
H	4.543384	3.325818	2.971175
H	3.070261	2.423105	3.371073
H	6.605420	1.766842	5.102325
H	7.273229	0.439890	4.112120
H	7.180126	2.078700	3.441700
H	4.346959	0.379714	5.682928
H	3.226199	-0.271631	4.450372
H	4.795918	-1.052825	4.716998
H	-1.080642	-1.847867	3.723467
H	0.044111	-2.656184	2.597145
H	0.673986	-1.862257	4.051660
H	-1.265161	0.868778	3.994941
H	0.475808	1.050047	4.342023
H	-0.273872	2.025860	3.065750
H	-2.247709	-0.377534	1.760086
H	-1.356801	0.713740	0.663685
H	-1.209460	-1.039919	0.465278

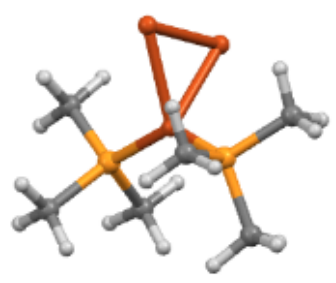

29

System = Cu3_PMe3; C6H18Cu3P2(2) ; E(SCF) = -1514.36482756 Hartree; G(298.150 K) = -1514.188557 Hartree; Convergence on forces and displacements = 2 YES; Minimal frequency = 16.7205 cm-1; S**2 = 0.7647

C	2.684461	-0.721198	-1.172283
P	2.779717	0.820935	-0.179959
C	4.202414	0.489332	0.936791
C	1.340881	0.659849	0.945484
Cu	2.789909	2.686275	-1.391235
Cu	0.884204	4.076373	-0.838100
Cu	0.730601	2.562686	-2.658500
P	4.237237	4.071449	-2.355188
C	3.482438	5.282430	-3.506400
C	5.568644	3.323587	-3.378283
C	5.175251	5.148937	-1.200639
H	6.211529	4.093155	-3.818379
H	6.174211	2.659516	-2.755751
H	5.116516	2.730360	-4.177549
H	5.839435	5.829171	-1.744111
H	4.467467	5.730601	-0.604166
H	5.767771	4.527419	-0.524103
H	4.235805	5.953302	-3.932146
H	2.974075	4.741404	-4.308789
H	2.733581	5.863396	-2.961609
H	4.071486	-0.453081	1.479057
H	5.121442	0.441218	0.346420
H	4.296000	1.308290	1.654962
H	1.346817	-0.303262	1.466424
H	1.364181	1.472782	1.675829
H	0.425232	0.758148	0.356380
H	2.640980	-1.605344	-0.527561
H	1.790266	-0.680013	-1.799954
H	3.559417	-0.789263	-1.824141

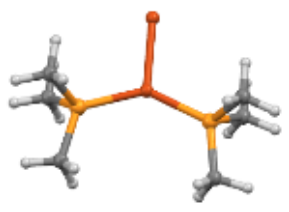

28

System = CuCu_2PMe3; C6H18Cu2P2 ; E(SCF) = -1316.95048409 Hartree; G(298.150 K) = -1316.771677 Hartree; Convergence on forces and displacements = 3 YES; Minimal frequency = 14.6493 cm-1; S**2 = None (restricted formalism)

C	3.363466	1.106040	-0.622734
P	2.308359	1.922192	-1.890825
C	0.795349	0.883610	-1.873628
Cu	3.081431	2.302577	-3.949399
P	4.916761	1.926559	-5.164149
C	5.668243	3.470599	-5.811286
Cu	1.490556	3.437952	-5.296918
C	1.762988	3.447076	-1.027829
C	6.370504	1.041573	-4.462477
C	4.572478	0.971779	-6.692910
H	0.396105	0.786057	-0.858860
H	0.044276	1.342911	-2.521435
H	1.029324	-0.109407	-2.266923
H	1.316977	3.212492	-0.055878
H	2.620083	4.110086	-0.881857
H	1.031080	3.963614	-1.654017
H	2.831769	0.991768	0.328022
H	3.667560	0.120519	-0.985885
H	4.263534	1.705722	-0.461675
H	7.176871	0.946868	-5.197670
H	6.740887	1.589230	-3.591545
H	6.062750	0.044420	-4.135858
H	6.508876	3.251445	-6.477620
H	4.903365	4.031240	-6.354772
H	6.018393	4.084571	-4.976992
H	5.466695	0.892499	-7.319593
H	4.227172	-0.030991	-6.426821
H	3.775834	1.474816	-7.247385

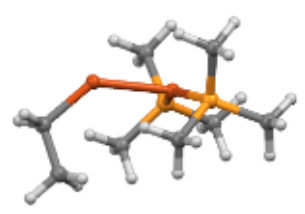

35

System = CuCu_Et_2PMe2; C8H23Cu2P2(2) ; E(SCF) = -1396.13800362 Hartree; G(298.150 K) = -1395.901191 Hartree; Convergence on forces and displacements = 4 YES; Minimal frequency = 24.2873 cm-1; S**2 = 0.7573

Cu	-0.244793	0.059991	-0.019423
Cu	-0.449432	-0.036670	2.405244
H	2.767650	0.732290	0.256817
C	2.612313	0.042767	-0.581814
C	1.256038	0.268586	-1.253619
H	3.462688	0.181237	-1.270500
H	2.697214	-0.971051	-0.172792
H	1.196185	1.284072	-1.674642
H	1.113680	-0.433707	-2.089228
P	-0.227442	2.110703	2.932470
C	0.030954	2.683965	4.663914
C	1.192657	2.861358	2.046498
C	-1.643330	3.139979	2.383474
H	1.238804	3.941009	2.221776
H	2.121827	2.398863	2.389660
H	1.082453	2.662336	0.977375
H	-1.450903	4.202470	2.564378
H	-1.805686	2.970323	1.315994
H	-2.545592	2.838480	2.922556
H	0.136595	3.773184	4.712757
H	-0.820491	2.377351	5.277584

H	0.932947	2.215850	5.067272
P	-0.206953	-2.186089	2.906625
C	0.133402	-2.768599	4.620714
C	-1.648571	-3.212130	2.421977
C	1.168010	-2.936271	1.951569
H	0.236227	-3.858524	4.658988
H	1.056210	-2.306396	4.981589
H	-0.685656	-2.461259	5.276576
H	1.231829	-4.013549	2.135765
H	0.997125	-2.752141	0.887933
H	2.111426	-2.463031	2.236113
H	-1.444529	-4.275700	2.582667
H	-2.521612	-2.918256	3.011051
H	-1.867618	-3.033760	1.366155

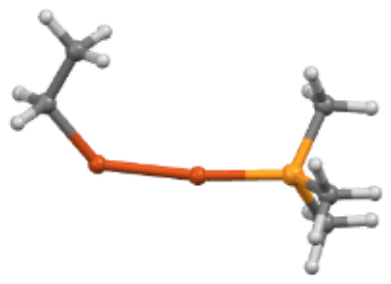

22
System = CuCu_Et_PMe3; C5H14Cu2P(2) ; E(SCF) = -935.055345933 Hartree; G(298.150 K) = -934.924613 Hartree; Convergence on forces and displacements = 4 YES; Minimal frequency = 11.9789 cm-1; S**2 = 0.7579

C	0.840127	0.042806	-0.015990
P	0.642386	-0.007196	1.805918
C	-1.159308	-0.260389	2.031876
Cu	1.492138	1.743682	2.901052
Cu	2.118188	3.735802	4.028255
C	1.272239	5.448621	4.422276
C	-0.112533	5.598839	3.804416
C	1.362583	-1.628593	2.267105
H	0.896720	-2.437388	1.695236
H	2.437508	-1.618023	2.069216
H	1.208323	-1.804638	3.334764
H	-1.503132	-1.135096	1.470601
H	-1.375917	-0.403497	3.093640
H	-1.696358	0.626210	1.685174
H	0.406408	-0.849567	-0.478606
H	0.343894	0.932950	-0.411047
H	1.902948	0.099074	-0.264877
H	1.240164	5.515348	5.518952
H	1.970940	6.211655	4.050609
H	-0.569791	6.573295	4.040105
H	-0.079794	5.516081	2.711938
H	-0.802031	4.826098	4.163434

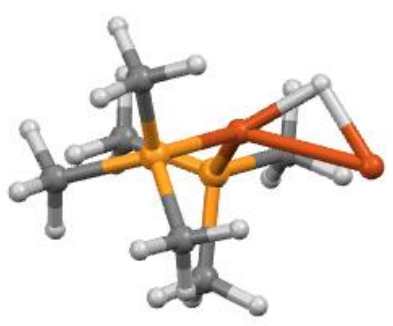

29
System = CuCu_H_2PMe2; C6H19Cu2P2(2) ; E(SCF) = -1317.53294578 Hartree; G(298.150 K) = -1317.348878 Hartree; Convergence on forces and displacements = 3 YES; Minimal frequency = 9.4620 cm-1; S**2 = 0.7553

Cu	0.476772	0.123178	-0.513122
Cu	0.847144	-0.072838	1.853035
H	1.780915	-0.181276	0.478773
P	0.300747	1.927940	2.710425
C	0.775809	2.250205	4.456870
C	0.926047	3.418792	1.841651
C	-1.512883	2.217979	2.737810
H	0.551132	4.339488	2.300827
H	2.019083	3.419592	1.866365

H	0.607207	3.375236	0.796566
H	-1.753275	3.200403	3.158086
H	-1.897209	2.153238	1.716334
H	-1.994430	1.439966	3.336290
H	0.427456	3.233655	4.789489
H	0.339420	1.478368	5.096336
H	1.863833	2.202702	4.553315
P	0.109388	-1.951317	2.827869
C	-0.445268	-1.826460	4.575894
C	-1.371819	-2.645881	1.993367
C	1.250832	-3.389034	2.877357
H	-0.827441	-2.784099	4.944838
H	0.392976	-1.508998	5.202147
H	-1.233694	-1.072298	4.648946
H	0.771400	-4.262198	3.332127
H	1.560209	-3.633722	1.857831
H	2.142516	-3.126101	3.452866
H	-1.715406	-3.560515	2.488417
H	-2.173281	-1.902690	2.003780
H	-1.123670	-2.863316	0.951268

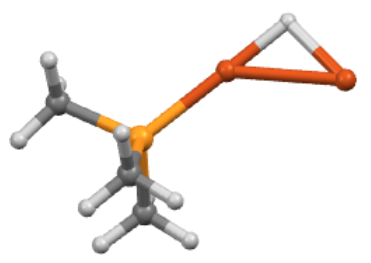

16

System = CuCu_H_PMe3; C3H10Cu2P(2) ; E(SCF) = -856.453126616 Hartree; G(298.150 K) = -856.371440 Hartree; Convergence on forces and displacements = 3 YES; Minimal frequency = 14.0963 cm-1; S**2 = 0.7548

C	0.627088	0.255773	-0.573662
P	0.680981	-0.025840	1.235493
C	2.429468	-0.466171	1.554826
Cu	-0.767050	-1.480134	2.047602
Cu	-1.254362	-2.400308	4.227442
C	0.518120	1.655841	1.941647
H	-1.811268	-2.524363	2.609826
H	1.271833	2.327138	1.518504
H	-0.478831	2.047127	1.723782
H	0.642823	1.605997	3.026163
H	2.586665	-0.562304	2.631934
H	2.655314	-1.425969	1.083443
H	1.387127	0.984188	-0.873168
H	0.804876	-0.688141	-1.095074
H	-0.361374	0.627610	-0.854948
H	3.099882	0.301000	1.155276

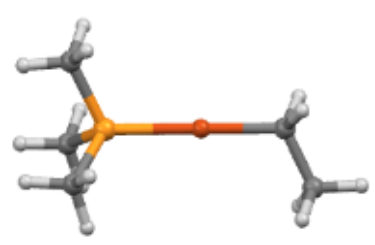

21

System = Cu_Et_PMe3; C5H14CuP ; E(SCF) = -737.677622816 Hartree; G(298.150 K) = -737.539036 Hartree; Convergence on forces and displacements = 4 YES; Minimal frequency = 23.0708 cm-1; S**2 = None (restricted formalism)

C	0.596439	-0.432944	2.448052
C	1.860065	0.421585	2.251465
Cu	-0.742966	-0.270408	1.062750
H	0.135586	-0.180398	3.417672
H	0.886860	-1.493127	2.537200
H	1.622874	1.491876	2.200291
H	2.376636	0.173190	1.315600
H	2.597090	0.298924	3.062336
P	-2.274789	-0.081084	-0.537740
C	-1.649472	-0.311433	-2.247190

C	-3.667743	-1.271379	-0.442009
C	-3.124206	1.542848	-0.624328
H	-3.856560	1.557360	-1.437738
H	-3.632646	1.738148	0.323455
H	-2.385283	2.331422	-0.789024
H	-4.375831	-1.110987	-1.261261
H	-3.278826	-2.291659	-0.494940
H	-4.185856	-1.148220	0.512652
H	-2.457527	-0.202193	-2.977509
H	-0.873599	0.430602	-2.453041
H	-1.208654	-1.307242	-2.341898

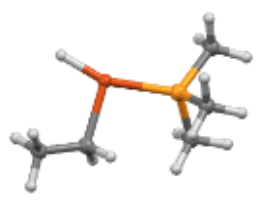

22
System = Cu_HEt_1P; C5H15CuP(2) ; E(SCF) = -738.227090119 Hartree; G(298.150 K) = -738.084482 Hartree; Convergence on forces and displacements = 4 YES; Minimal frequency = 31.9003 cm-1; S**2 = 0.7550

C	4.054128	1.928574	2.351180
P	3.537200	1.447173	0.659945
C	5.096156	0.847861	-0.096296
C	2.580194	-0.093864	0.933445
Cu	2.563422	3.140085	-0.528161
C	3.887956	4.645061	-0.056002
C	3.709424	5.918604	-0.844406
H	1.555277	3.835651	-1.459528
H	4.555487	1.098386	2.858322
H	4.737192	2.779682	2.291727
H	3.176721	2.228839	2.929891
H	5.790046	1.684870	-0.209030
H	5.560727	0.075736	0.524934
H	4.886718	0.436702	-1.087195
H	1.661297	0.141057	1.477047
H	2.308314	-0.528008	-0.032204
H	3.163215	-0.822136	1.506547
H	3.681996	4.751473	1.015393
H	4.858544	4.160525	-0.213089
H	4.442400	6.686867	-0.547401
H	3.837027	5.748402	-1.918710
H	2.711824	6.344471	-0.696437

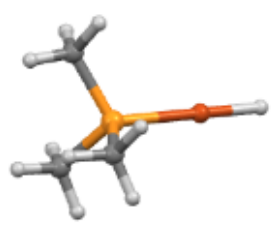

15
System = Cu_H_PMe3; C3H10CuP ; E(SCF) = -659.067118043 Hartree; G(298.150 K) = -658.979845 Hartree; Convergence on forces and displacements = 4 YES; Minimal frequency = 113.2262 cm-1; S**2 = None (restricted formalism)

Cu	-0.301356	0.076146	0.525346
H	-1.671452	0.121631	1.199758
P	1.692558	0.010012	-0.461310
C	1.650736	0.037209	-2.293956
C	2.815634	1.397179	-0.042190
C	2.698355	-1.475779	-0.084776
H	3.772704	1.292604	-0.562918
H	2.349196	2.343196	-0.328994
H	2.990887	1.410690	1.036662
H	3.661559	-1.433351	-0.602840
H	2.869818	-1.536713	0.992956
H	2.157055	-2.371400	-0.400350
H	2.663938	0.002015	-2.706351
H	1.082354	-0.822814	-2.657517
H	1.153400	0.949378	-2.633408

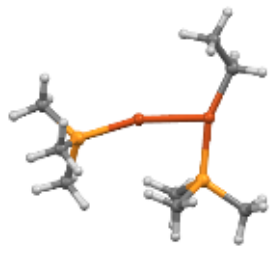

35

System = CuPMe3_Cu_Et_PMe3; C8H23Cu2P2(2) ; E(SCF) = -1396.13095272 Hartree; G(298.150 K) = -1395.893823 Hartree; Convergence on forces and displacements = 4 YES; Minimal frequency = 23.0819 cm-1; S**2 = 0.7539

C	-0.589588	2.462097	0.869726
P	0.080469	1.068299	1.860509
Cu	-1.255818	0.338917	3.534727
Cu	-2.848074	0.213006	5.421016
P	-3.250835	2.356333	4.890835
C	-3.903411	2.618494	3.191970
C	1.717833	1.699211	2.399011
C	0.512656	-0.175094	0.582410
C	-1.693957	-2.336214	6.450600
C	-2.982747	-1.512429	6.335064
C	-4.466790	3.319903	5.896079
C	-1.788795	3.471025	4.941487
H	-1.278330	-2.564860	5.460795
H	-1.832455	-3.297008	6.975274
H	-0.911768	-1.788681	6.992404
H	-3.747352	-2.103024	5.802629
H	-3.396255	-1.340544	7.343794
H	2.309966	2.036823	1.542160
H	1.578156	2.533396	3.091627
H	2.257498	0.905019	2.921536
H	1.161568	0.258073	-0.185721
H	1.024702	-1.016982	1.055839
H	-0.403012	-0.547000	0.115089
H	0.122087	2.773472	0.098183
H	-1.523095	2.152687	0.392193
H	-0.801255	3.310287	1.526167
H	-4.061372	3.682963	2.990276
H	-3.196591	2.208899	2.466117
H	-4.852136	2.085584	3.084236
H	-2.064344	4.494230	4.665801
H	-1.367841	3.469194	5.950660
H	-1.025482	3.098809	4.253588
H	-4.571228	4.347168	5.528405
H	-5.439343	2.821763	5.856889
H	-4.133996	3.341518	6.937449

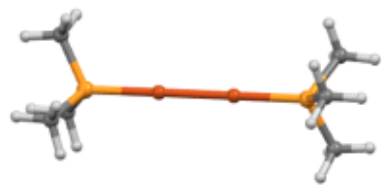

28

System = CuPMe3_CuPMe3; C6H18Cu2P2 ; E(SCF) = -1316.94648439 Hartree; G(298.150 K) = -1316.769238 Hartree; Convergence on forces and displacements = 3 YES; Minimal frequency = 13.9012 cm-1; S**2 = None (restricted formalism)

C	5.233957	5.563492	4.391909
P	4.774160	4.497993	2.966131
C	3.780273	5.640900	1.924258
C	3.485633	3.405195	3.690905
Cu	6.517061	3.454702	1.907786
Cu	8.308792	2.423990	0.823393
P	10.071234	1.410066	-0.231303
C	9.768715	0.860182	-1.959333
C	11.595025	2.424411	-0.401464
C	10.703490	-0.121778	0.564655
H	5.926026	6.340346	4.055547
H	4.351122	6.034300	4.837086

H	5.739666	4.957450	5.148585
H	3.352377	5.086379	1.084618
H	4.430262	6.423377	1.523282
H	2.972238	6.102989	2.501192
H	2.695818	3.989650	4.174698
H	3.945836	2.741567	4.428009
H	3.046181	2.789531	2.901344
H	12.387756	1.867989	-0.912565
H	11.365922	3.329793	-0.970138
H	11.945344	2.721766	0.590729
H	10.658845	0.387098	-2.387461
H	8.939736	0.147304	-1.972874
H	9.489584	1.722754	-2.570576
H	11.544480	-0.543378	0.003978
H	11.027825	0.106530	1.583596
H	9.899304	-0.860726	0.618068

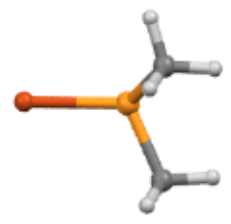

14

System = Cu_PMe3; C3H9CuP(2) ; E(SCF) = -658.428429477 Hartree; G(298.150 K) = -658.348687 Hartree; Convergence on forces and displacements = 4 YES; Minimal frequency = 86.4916 cm-1; S**2 = 0.7534

Cu	-0.160850	0.000000	-0.567771
P	-0.119245	0.000000	1.702430
C	1.555434	-0.000000	2.452587
C	-0.934501	-1.437962	2.499369
C	-0.934501	1.437962	2.499369
H	1.495828	-0.000000	3.545818
H	2.101984	-0.886758	2.120736
H	2.101984	0.886758	2.120736
H	-0.876976	-1.366571	3.590360
H	-1.983721	-1.475890	2.194740
H	-0.446611	-2.359709	2.171583
H	-0.876976	1.366571	3.590360
H	-0.446611	2.359709	2.171583
H	-1.983721	1.475890	2.194740

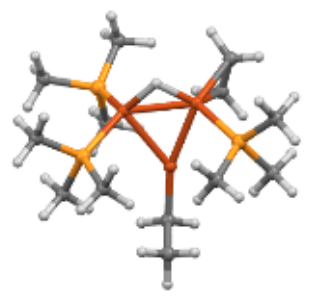

56

System = IR1_mix; C13H37Cu3P3(2) ; E(SCF) = -2133.85348797 Hartree; G(298.150 K) = -2133.458732 Hartree; Convergence on forces and displacements = 3 YES; Minimal frequency = 24.1538 cm-1; S**2 = 0.7582

Cu	0.973339	0.843102	2.480136
Cu	-0.867771	0.216483	0.937323
C	-2.646367	0.016169	0.173044
H	-3.309754	-0.256486	1.008977
H	-2.625389	-0.852968	-0.502435
C	-3.189124	1.248253	-0.552432
H	-2.558136	1.531869	-1.403940
H	-4.206976	1.101507	-0.951088
H	-3.234307	2.121088	0.110715
P	-0.203049	2.740887	2.887244
C	0.461745	3.993468	4.068550
C	-0.575047	3.780354	1.419750
C	-1.886341	2.452145	3.570673
H	0.618585	3.522725	5.043259
H	1.427885	4.353711	3.704093
H	-0.220399	4.843237	4.184813
H	0.359622	4.070718	0.933473

H	-1.139795	4.677637	1.694499
H	-1.158342	3.184094	0.713971
H	-2.431882	3.391836	3.708200
H	-2.436309	1.805095	2.880998
H	-1.805818	1.940056	4.533883
P	1.791972	-2.332224	0.311976
C	2.672974	-3.136194	-1.092525
C	0.170840	-3.198090	0.275118
C	2.624215	-3.114384	1.753494
H	-0.422774	-2.887133	1.138674
H	0.293141	-4.286603	0.282027
H	-0.373020	-2.895938	-0.623790
H	3.702586	-2.769728	-1.131067
H	2.682635	-4.227104	-0.992686
H	2.180368	-2.868839	-2.031367
H	3.662636	-2.774161	1.793646
H	2.131104	-2.801444	2.676680
H	2.603174	-4.207384	1.686002
P	1.162419	0.698249	-1.774328
C	2.559889	0.711379	-2.975014
C	-0.104330	-0.273874	-2.683933
C	0.509699	2.406250	-1.934899
H	-0.306246	0.153388	-3.671859
H	0.244420	-1.303345	-2.803273
H	-1.023111	-0.289237	-2.092784
H	0.310539	2.665554	-2.980173
H	-0.414672	2.480653	-1.358287
H	1.232575	3.110662	-1.515207
H	2.238033	1.061845	-3.961858
H	3.352175	1.365759	-2.601055
H	2.966237	-0.299584	-3.067412
Cu	1.580150	-0.082344	0.294943
C	0.241590	-0.752589	3.769166
C	1.355592	-0.213789	4.376090
H	0.302154	-1.664911	3.186266
H	-0.759590	-0.431191	4.041510
H	2.338119	-0.659005	4.249507
H	1.265392	0.570524	5.122579
H	2.288958	0.840994	1.491383

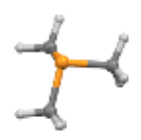

13

System = PMe3; C3H9P ; E(SCF) = -461.041956152 Hartree; G(298.150 K) = -460.958354 Hartree; Convergence on forces and displacements = 4 YES; Minimal frequency = 179.6671 cm-1; S**2 = None (restricted formalism)

P	-0.080447	-0.128359	-0.115837
C	0.044059	0.054333	1.723746
C	1.714746	-0.014782	-0.559960
C	-0.618034	1.587607	-0.562281
H	-0.957805	0.161954	2.150693
H	0.497769	-0.844806	2.151914
H	0.646249	0.925264	2.008547
H	1.820984	0.040486	-1.647734
H	2.191819	0.865047	-0.112009
H	2.235728	-0.913563	-0.215926
H	-0.599419	1.706603	-1.649994
H	-1.645680	1.750552	-0.223213
H	0.029214	2.349103	-0.111247

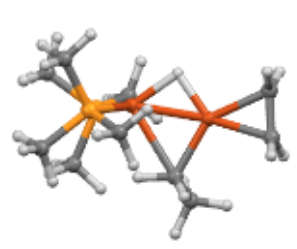

42

System = prod_TS1_2PMe3; C10H28Cu2P2 ; E(SCF) = -1475.36979851 Hartree; G(298.150 K) = -1475.073811 Hartree; Convergence on forces and displacements = 4 YES; Minimal frequency = 18.2954 cm-1; S**2 = None (restricted formalism)

C	0.825963	3.382484	2.899620
P	1.201840	2.324969	1.443641
C	2.883786	2.901840	0.975874
C	0.156341	3.083269	0.136083
Cu	0.989591	0.135277	1.833588
C	-1.101910	-0.020787	3.197052
C	-2.222292	1.019928	3.082924
P	2.504947	-0.811210	3.192964
C	3.097802	0.216116	4.598770
Cu	-0.981819	-1.128198	1.505256
C	-2.681752	-2.198279	1.477650
C	-1.944593	-2.498842	0.333549
C	1.897289	-2.316833	4.051736
C	4.082455	-1.385545	2.446082
H	0.384128	-0.869786	0.610890
H	-3.504139	-1.486808	1.442581
H	-2.664367	-2.856398	2.343829
H	-1.332648	-3.396392	0.281531
H	-2.171368	-2.024069	-0.618206
H	-0.205616	0.491259	3.601803
H	-1.350541	-0.757303	3.975725
H	-2.383749	1.598241	4.007957
H	-2.015256	1.747305	2.287908
H	-3.181015	0.551933	2.828484
H	0.943251	4.446037	2.666331
H	-0.199930	3.193764	3.225844
H	1.498739	3.115074	3.718784
H	2.911689	3.987335	0.833151
H	3.590621	2.623784	1.762620
H	3.191377	2.408735	0.049683
H	0.329390	4.161756	0.054198
H	0.378418	2.606044	-0.822267
H	-0.896176	2.902047	0.369704
H	2.649477	-2.732557	4.730683
H	0.997022	-2.060762	4.616885
H	1.625791	-3.066795	3.303873
H	4.735055	-1.849953	3.193021
H	3.863551	-2.109447	1.656497
H	4.599564	-0.533641	1.995920
H	3.787506	-0.337326	5.245108
H	3.604827	1.102884	4.208142
H	2.237948	0.545698	5.188827

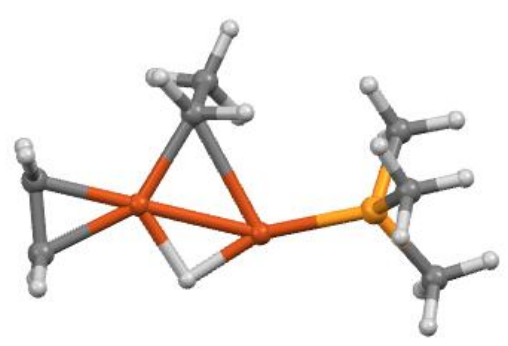

29

System = prod_TS1CuI; C7H19Cu2P ; E(SCF) = -1014.28799998 Hartree; G(298.150 K) = -1014.097520 Hartree; Convergence on forces and displacements = 2 YES; Minimal frequency = 17.5374 cm-1; S**2 = None (restricted formalism)

Cu	2.085096	1.557422	0.250227
C	4.367357	2.771757	0.411862
Cu	4.248600	0.791659	0.775154
C	5.217191	-0.935669	1.327460
C	6.100321	0.133172	1.229699
H	2.700984	0.120298	0.701012
H	5.110660	-1.652422	0.516731
H	4.814719	-1.240741	2.290259

H	6.712048	0.280117	0.342487
H	6.411195	0.682638	2.115727
C	4.201234	3.590512	1.699680
H	4.894279	3.252165	2.479939
H	5.385484	2.904564	0.017022
H	3.721045	3.202422	-0.372944
H	4.375639	4.669938	1.560870
P	0.596735	3.053662	-0.372445
C	0.656307	3.432907	-2.165236
H	1.657433	3.785695	-2.426398
H	-0.078277	4.201411	-2.425124
C	-1.172099	2.676128	-0.078670
H	-1.437427	1.752775	-0.599518
H	-1.808904	3.491005	-0.437046
H	-1.339022	2.530091	0.991608
H	0.685696	4.606616	1.490473
C	0.814279	4.697505	0.408963
H	1.826676	5.058441	0.211771
H	0.087561	5.415497	0.016495
H	3.191925	3.485495	2.120541
H	0.448512	2.525275	-2.737434

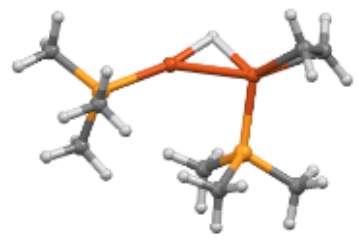

35

System = prod_TS1Cu; C8H23Cu2P2(2) ; E(SCF) = -1396.13064812 Hartree; G(298.150 K) = -1395.896346 Hartree; Convergence on forces and displacements = 4 YES; Minimal frequency = 25.0191 cm-1; S**2 = 0.7549

C	-0.513125	2.494302	0.988499
P	0.097713	0.921926	1.710351
Cu	-1.317507	-0.159917	3.012958
Cu	-2.569599	-0.225101	5.044648
P	-3.072933	1.945054	4.810750
C	-3.845600	2.463129	3.222788
C	1.644186	1.426814	2.556455
C	0.708100	0.006009	0.242954
C	-1.956887	-1.100743	6.848534
C	-3.358691	-1.216830	6.716495
C	-4.274756	2.602718	6.042525
C	-1.706285	3.171609	4.974407
H	-2.388089	-1.173693	3.648694
H	-1.309854	-1.945661	6.626454
H	-1.525808	-0.327448	7.480472
H	-3.805765	-2.151533	6.386197
H	-4.020548	-0.531871	7.241459
H	2.306427	1.965315	1.871118
H	1.402921	2.067627	3.408139
H	2.155845	0.537031	2.932161
H	1.423817	0.610249	-0.323779
H	1.192474	-0.918048	0.568652
H	-0.136772	-0.253682	-0.399884
H	0.265988	2.967956	0.383027
H	-1.384435	2.293047	0.359990
H	-0.810883	3.179446	1.785825
H	-4.052081	3.538761	3.205015
H	-3.177113	2.207733	2.396272
H	-4.779216	1.911650	3.083843
H	-2.069189	4.201430	4.880560
H	-1.226253	3.042641	5.948336
H	-0.957077	2.976951	4.203600
H	-4.467207	3.670108	5.887798

H	-5.213601	2.048846	5.960251
H	-3.875134	2.451508	7.049260

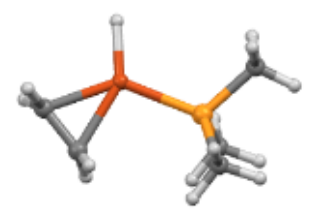

21

System = prod_TS1; C5H14CuP ; E(SCF) = -737.652576078 Hartree; G(298.150 K) = -737.517067 Hartree; Convergence on forces and displacements = 3 YES; Minimal frequency = 28.3084 cm-1; S**2 = None (restricted formalism)

C	-2.721050	-0.223656	-2.446906
P	-1.922799	-0.105728	-0.800393
C	-3.019998	1.055584	0.103532
Cu	0.240668	0.544729	-0.790279
C	-2.330356	-1.724352	-0.036377
C	1.838815	0.580712	0.503970
C	0.765646	-0.023955	1.137739
H	2.071667	1.629416	0.670039
H	2.620218	-0.011507	0.034574
H	0.715961	1.219652	-2.112901
H	0.679325	-1.107132	1.181440
H	0.130855	0.535335	1.821314
H	-4.063571	0.728992	0.052613
H	-2.709532	1.108769	1.150331
H	-2.933006	2.053352	-0.334465
H	-3.404420	-1.929182	-0.091545
H	-1.786308	-2.519057	-0.554051
H	-2.017728	-1.718122	1.011090
H	-3.777458	-0.497501	-2.359750
H	-2.634266	0.739972	-2.955625
H	-2.200514	-0.974602	-3.047229

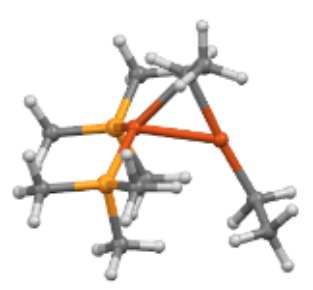

42

System = reac_but; C10H28Cu2P2 ; E(SCF) = -1475.38616929 Hartree; G(298.150 K) = -1475.087374 Hartree; Convergence on forces and displacements = 2 YES; Minimal frequency = 19.5979 cm-1; S**2 = None (restricted formalism)

Cu	1.086574	0.268293	-0.668970
Cu	2.224210	-0.292772	1.331967
C	1.566914	-1.425533	2.756495
C	0.946462	-0.676542	3.947748
H	2.373159	-2.082900	3.125348
H	0.813651	-2.111511	2.331879
H	0.112192	-0.035152	3.634393
H	0.551279	-1.343621	4.732739
H	1.674961	-0.014790	4.433572
C	3.025332	0.988750	-0.027361
C	3.264921	2.292499	0.750136
H	3.974937	0.443392	-0.155049
H	2.778874	1.268662	-1.079912
H	4.032775	2.934589	0.291602
H	2.345682	2.883626	0.820686
H	3.589437	2.094819	1.780015
P	1.083224	-1.561596	-1.944484
C	2.709866	-2.092523	-2.610015
C	0.577366	-3.010833	-0.938109
C	-0.015365	-1.691990	-3.412747
H	2.641795	-3.063012	-3.112686
H	3.077514	-1.344300	-3.317565
H	3.420907	-2.160654	-1.782225

H	0.058206	-2.678566	-3.882373
H	-1.049370	-1.516775	-3.102622
H	0.258369	-0.925188	-4.142724
H	0.623025	-3.937239	-1.519964
H	1.239109	-3.083720	-0.071306
H	-0.441842	-2.860567	-0.573221
P	-0.774325	1.210989	0.114070
C	-1.752238	-0.100004	0.949321
C	-0.575974	2.457991	1.446657
C	-2.022594	1.997350	-0.983183
H	-1.537825	2.707832	1.906556
H	0.099374	2.054553	2.205756
H	-0.127096	3.365545	1.034400
H	-2.317853	1.286432	-1.759656
H	-2.909685	2.305599	-0.419817
H	-1.582730	2.873235	-1.467989
H	-2.621255	0.317727	1.468175
H	-2.090294	-0.825319	0.204246
H	-1.107738	-0.616473	1.665077

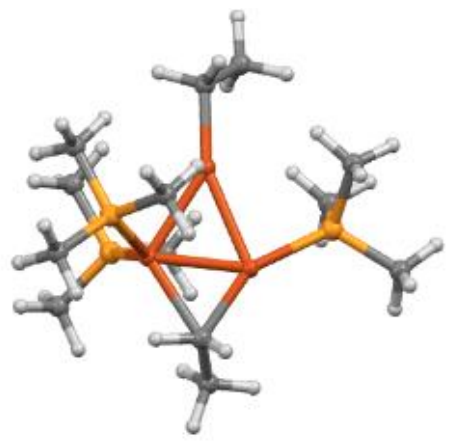

56

System = reac_TS1_mix; C13H37Cu3P3(2) ; E(SCF) = -2133.87203898 Hartree; G(298.150 K) = -2133.472502 Hartree; Convergence on forces and displacements = 3 YES; Minimal frequency = 14.3957 cm-1; S**2 = 0.7555

Cu	0.880481	1.020821	2.194434
Cu	-0.953447	0.127481	0.648617
C	-2.750904	-0.099714	-0.100379
H	-3.319782	-0.754438	0.579819
H	-2.641009	-0.660829	-1.042704
C	-3.526401	1.195899	-0.354590
H	-2.984415	1.864476	-1.036338
H	-4.522537	1.031321	-0.799869
H	-3.688674	1.760022	0.572682
P	-0.671121	2.382605	2.984191
C	-0.331759	3.523771	4.394910
C	-1.256171	3.548838	1.692662
C	-2.222588	1.564924	3.526545
H	-0.064579	2.937700	5.278592
H	0.513789	4.169258	4.141551
H	-1.204420	4.145488	4.624923
H	-0.421956	4.173002	1.361168
H	-2.058849	4.188224	2.074437
H	-1.621642	2.971271	0.840247
H	-2.972165	2.304804	3.826018
H	-2.602821	0.961235	2.697489
H	-2.009614	0.902174	4.369871
P	0.937369	-2.630242	0.703748
C	2.218546	-3.957746	0.675733
C	-0.271596	-3.245998	-0.534655
C	0.075502	-2.984644	2.287696
H	-1.134160	-2.574176	-0.540276
H	-0.597195	-4.265716	-0.303175
H	0.185780	-3.229256	-1.527569
H	2.952916	-3.765334	1.463150
H	1.777705	-4.949033	0.829765
H	2.736381	-3.939562	-0.287383
H	0.736655	-2.728818	3.120433

H	-0.808824	-2.343732	2.345451
H	-0.220089	-4.036746	2.364277
P	1.624381	0.597639	-1.479519
C	3.203000	0.694561	-2.430163
C	0.452592	-0.017043	-2.754585
C	1.150321	2.370457	-1.367434
H	0.469063	0.611542	-3.651338
H	0.716544	-1.042438	-3.026750
H	-0.552909	-0.021986	-2.324761
H	1.201438	2.867376	-2.342359
H	0.130853	2.423162	-0.975891
H	1.811023	2.880178	-0.661104
H	3.085188	1.272956	-3.353382
H	3.970492	1.163682	-1.808078
H	3.536388	-0.316946	-2.678648
Cu	1.472903	-0.481354	0.464015
C	2.805123	0.419549	2.075424
C	3.687881	1.637075	1.773057
H	3.214519	-0.450158	1.515666
H	2.919775	0.110678	3.126307
H	4.757751	1.452206	1.963154
H	3.400266	2.507700	2.374805
H	3.599209	1.938979	0.723361

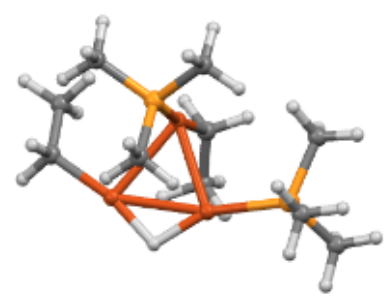

44

System = reac_TS2CuI; C10H29Cu3P2 ; E(SCF) = -1673.42296182 Hartree; G(298.150 K) = -1673.115880 Hartree; Convergence on forces and displacements = 4 YES; Minimal frequency = 28.0588 cm-1; S**2 = None (restricted formalism)

Cu	2.028171	1.034149	-1.071088
Cu	2.871200	0.822513	1.255490
H	3.407533	1.132597	-0.221127
Cu	0.481996	0.370719	0.898430
C	0.639451	0.990367	-2.424394
C	-0.779574	1.334817	-1.954514
H	0.935993	1.696582	-3.218379
H	0.639390	0.000567	-2.910138
H	-1.117545	0.659655	-1.155044
H	-1.534928	1.282310	-2.756338
H	-0.825565	2.345630	-1.532378
P	3.176189	0.182181	3.345764
C	4.083489	-1.402479	3.518454
C	4.140972	1.333158	4.397812
C	1.658980	-0.095338	4.332700
H	3.534733	-2.197220	3.007293
H	5.067467	-1.309190	3.051900
H	4.205080	-1.670442	4.572746
H	5.129332	1.487222	3.957615
H	3.627115	2.296963	4.441618
H	4.252135	0.935758	5.411748
H	1.006303	-0.798088	3.809866
H	1.904634	-0.484413	5.325789
H	1.122236	0.850607	4.432232
P	0.575863	-1.769398	0.286597
C	0.240859	-2.986418	1.621609
C	-0.598283	-2.276683	-1.025538
C	2.191884	-2.330474	-0.370703
H	-0.750746	-2.802163	2.043151
H	0.980511	-2.872126	2.417754
H	0.285912	-4.010912	1.238203

H	-0.472251	-3.335472	-1.272848
H	-0.418830	-1.668562	-1.914774
H	-1.622688	-2.103086	-0.686143
H	2.974493	-2.139085	0.367703
H	2.424244	-1.750072	-1.267593
H	2.166315	-3.397611	-0.613237
C	0.276378	2.148869	1.661390
H	0.125650	2.037850	2.745245
H	-0.703758	2.443100	1.249282
C	1.277615	3.276336	1.395924
H	1.410825	3.447166	0.320827
H	0.980350	4.235269	1.848929
H	2.278665	3.043906	1.797487

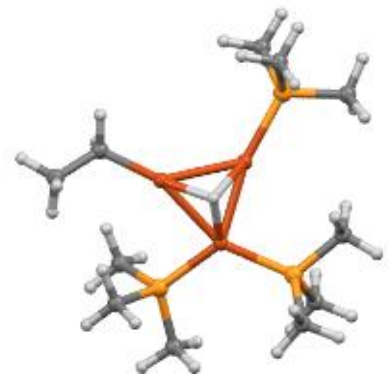

50

System = reac_TS2Cu; C11H33Cu3P3(2) ; E(SCF) = -2055.26168345 Hartree; G(298.150 K) = -2054.915593 Hartree; Convergence on forces and displacements = 4 YES; Minimal frequency = 16.1599 cm-1; S**2 = 0.7545

C	-0.992863	-2.219081	3.405393
P	-0.432089	-0.531706	2.942258
C	-2.004985	0.424684	3.034358
Cu	0.711768	-0.443830	1.027067
Cu	2.865909	-0.121134	1.988556
P	4.590196	0.328213	3.300236
C	5.186492	2.065827	3.345279
Cu	2.550292	0.461473	-0.391380
C	3.640801	0.362299	-1.995783
C	2.958114	0.755314	-3.314744
C	0.429028	0.017435	4.470579
C	6.102807	-0.605151	2.846379
C	4.378886	-0.071529	5.079182
H	1.774279	0.921183	1.010942
H	4.043766	-0.659638	-2.087139
H	4.522883	1.006316	-1.842838
H	2.558317	1.776841	-3.277447
H	2.109577	0.098913	-3.546364
H	3.633385	0.710002	-4.185731
H	6.064476	2.168742	3.992012
H	5.443670	2.381655	2.330874
H	4.385998	2.712426	3.714240
H	6.933479	-0.358766	3.515743
H	5.895170	-1.676859	2.901209
H	6.380275	-0.361076	1.817582
H	5.299087	0.128968	5.637432
H	3.568010	0.532281	5.494881
H	4.114804	-1.127117	5.184245
H	-0.227591	-0.045354	5.345083
H	1.305413	-0.616798	4.626760
H	0.768781	1.048819	4.343499
H	-2.507586	0.282727	3.997676
H	-1.788009	1.486892	2.892567
H	-2.669901	0.100493	2.229232
H	-1.536942	-2.211417	4.355877
H	-1.644529	-2.610897	2.619908
H	-0.121516	-2.873790	3.488147
P	-0.210410	-1.159469	-0.872768
C	0.811346	-2.286342	-1.899347
C	-1.767853	-2.121030	-0.683972

C	-0.700903	0.142439	-2.073048
H	-1.127771	-0.291600	-2.983690
H	-1.435971	0.803124	-1.605597
H	0.181298	0.734795	-2.328663
H	-2.162152	-2.438942	-1.655046
H	-1.572344	-3.004462	-0.070127
H	-2.515383	-1.505842	-0.175909
H	0.289476	-2.574974	-2.818179
H	1.748315	-1.779706	-2.143574
H	1.048591	-3.179857	-1.315753

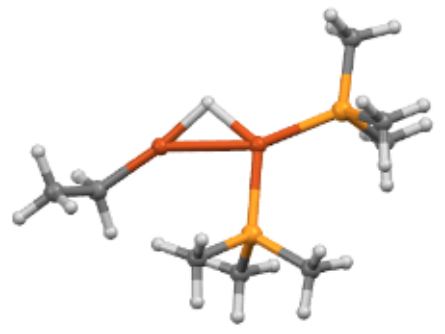

36

System = reac_TS2; C8H24Cu2P2 ; E(SCF) = -1396.78009076 Hartree; G(298.150 K) = -1396.533051 Hartree; Convergence on forces and displacements = 4 YES; Minimal frequency = 21.9803 cm-1; S**2 = None (restricted formalism)

C	3.872539	3.987646	3.890111
P	4.318566	2.430844	3.027326
C	5.106230	3.029745	1.482848
Cu	1.586445	2.666175	1.227006
Cu	2.554075	1.029811	2.830056
C	1.681225	4.450565	0.464013
C	2.178026	4.519007	-0.990227
C	5.765762	1.842511	3.999539
H	1.319963	1.157233	1.754775
H	2.337258	5.074997	1.095093
H	0.690516	4.932024	0.524903
H	3.178963	4.080798	-1.102002
H	2.239601	5.546704	-1.386568
H	1.521399	3.958553	-1.667808
H	3.483217	3.754168	4.884726
H	4.740261	4.648319	3.986370
H	3.089390	4.491774	3.318326
H	6.185749	0.952365	3.523036
H	5.443761	1.574716	5.009506
H	6.541569	2.612725	4.064551
H	5.927625	3.720407	1.700320
H	4.346021	3.534835	0.881794
H	5.489789	2.177803	0.914901
P	2.458054	-0.657836	4.315330
C	2.378999	-0.082495	6.058441
C	1.060952	-1.845252	4.224684
C	3.923114	-1.765666	4.329105
H	2.359487	-0.927722	6.754140
H	3.250110	0.541539	6.274543
H	1.479034	0.521652	6.200903
H	3.826325	-2.540140	5.097015
H	4.029561	-2.241215	3.350419
H	4.821195	-1.174387	4.524555
H	1.125366	-2.600889	5.014614
H	0.118880	-1.298932	4.321328
H	1.070968	-2.339318	3.249406

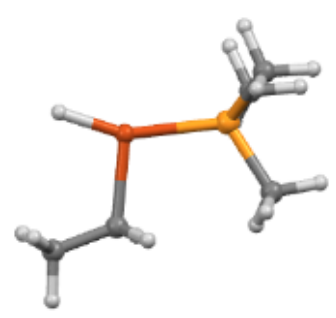

22

System = reac_TS3; C5H15CuP(2) ; E(SCF) = -738.226673431 Hartree; G(298.150 K) = -738.084252 Hartree; Convergence on forces and displacements = 4 YES; Minimal frequency = 24.0744 cm-1; S**2 = 0.7550

Cu	2.431121	3.500225	-1.585327
H	2.433519	4.543335	-2.717225
C	3.168218	4.552097	0.018087
H	2.338898	4.443855	0.727066
H	4.007635	3.911801	0.311916
C	3.554226	5.981960	-0.265048
H	4.330928	6.042859	-1.034608
H	2.698065	6.566866	-0.616670
H	3.946013	6.483943	0.635111
P	2.063250	1.444243	-0.643082
C	2.779070	1.203649	1.026991
C	2.712227	0.007874	-1.581732
C	0.293887	1.025512	-0.402000
H	2.389706	1.967526	1.704693
H	3.865804	1.310189	0.975948
H	2.531528	0.212318	1.419044
H	3.793474	0.112659	-1.704075
H	2.253245	-0.015393	-2.573448
H	2.497082	-0.930313	-1.060302
H	0.185010	0.035302	0.051802
H	-0.217788	1.038617	-1.367840
H	-0.171651	1.772953	0.245812

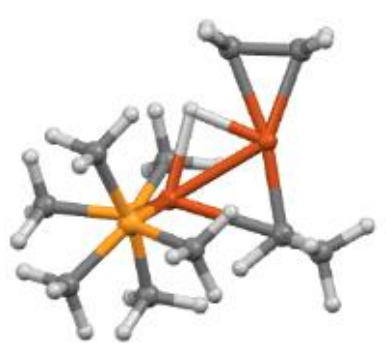

42

System = TS1_2PMe3; C10H28Cu2P2 ; E(SCF) = -1475.33850533 Hartree; G(298.150 K) = -1475.044244 Hartree; Convergence on forces and displacements = 3 YES; Minimal frequency = -869.4926 cm-1; S**2 = None (restricted formalism)

C	0.555579	3.729883	2.757162
P	0.933973	2.390629	1.557344
C	2.477822	2.995545	0.765797
C	-0.315036	2.672137	0.240671
Cu	0.918434	0.308499	2.346006
C	-1.184791	0.071327	3.599851
C	-2.331599	1.090923	3.603091
P	2.441677	-0.919011	3.415336
C	3.875617	-0.145561	4.268635
Cu	-1.062077	-0.898309	1.903353
C	-1.883438	-2.095216	0.507220
C	-0.546786	-1.734698	0.063636
C	1.717049	-1.969389	4.735423
C	3.253738	-2.158700	2.330569
H	0.322700	-0.828003	0.924642
H	-2.730947	-1.636415	0.003038
H	-2.048483	-3.109547	0.863363
H	0.207422	-2.521279	0.048195
H	-0.465381	-1.070483	-0.796437
H	-0.279194	0.588458	3.998747
H	-1.373470	-0.709423	4.350962
H	-2.448243	1.602307	4.572231
H	-2.181633	1.871186	2.847913
H	-3.289966	0.613441	3.369425
H	0.516971	4.707822	2.265806
H	-0.406989	3.525062	3.232495
H	1.324999	3.745162	3.533840
H	2.351652	4.007386	0.366311
H	3.285397	2.996664	1.502867

H	2.755228	2.319469	-0.047586
H	-0.303862	3.707709	-0.115133
H	-0.108107	1.997821	-0.594693
H	-1.306964	2.430949	0.631501
H	2.469378	-2.626075	5.184586
H	1.283469	-1.330677	5.509368
H	0.913695	-2.572379	4.304126
H	3.934510	-2.802157	2.897639
H	2.482980	-2.772323	1.856899
H	3.813002	-1.643576	1.544801
H	4.517870	-0.897154	4.740150
H	4.461917	0.424975	3.542976
H	3.509485	0.545470	5.033068

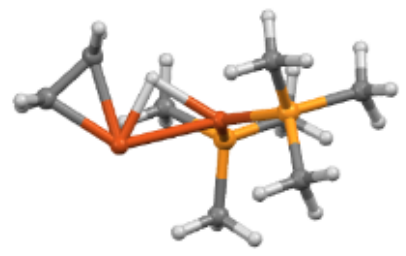

35

System = TS1Cu2P; C8H23Cu2P2(2) ; E(SCF) = -1396.08886146 Hartree; G(298.150 K) = -1395.858553 Hartree; Convergence on forces and displacements = 2 YES; Minimal frequency = -1127.7350 cm-1; S**2 = 0.7586

Cu	0.182413	-0.257380	-0.452563
Cu	0.246630	0.201547	1.940299
H	1.501369	0.156311	0.564236
C	2.331659	-0.234751	-0.614745
C	1.618901	-0.678961	-1.806439
H	2.837060	0.730203	-0.655278
H	2.897423	-0.979333	-0.055222
H	1.608357	-0.008656	-2.663634
H	1.672389	-1.736635	-2.056925
P	-0.060290	2.177769	2.934277
C	0.074331	2.323856	4.763426
C	1.048247	3.521173	2.352702
C	-1.729747	2.869008	2.605803
H	0.796296	4.476918	2.824012
H	2.084872	3.264781	2.587896
H	0.954788	3.614000	1.267560
H	-1.839578	3.863497	3.050670
H	-1.883379	2.932707	1.525493
H	-2.488142	2.200435	3.022015
H	-0.124488	3.347378	5.098962
H	-0.642693	1.644495	5.232162
H	1.080262	2.031606	5.077087
P	0.012976	-1.901593	2.697705
C	0.266539	-2.326600	4.471865
C	-1.653312	-2.597172	2.363715
C	1.118359	-3.096641	1.847361
H	0.114419	-3.395937	4.655126
H	1.282721	-2.052490	4.768697
H	-0.435966	-1.752951	5.082755
H	0.925654	-4.123925	2.173759
H	0.955135	-3.018598	0.768843
H	2.160638	-2.840533	2.056218
H	-1.712006	-3.647422	2.668036
H	-2.405620	-2.019606	2.907794
H	-1.860891	-2.511320	1.293805

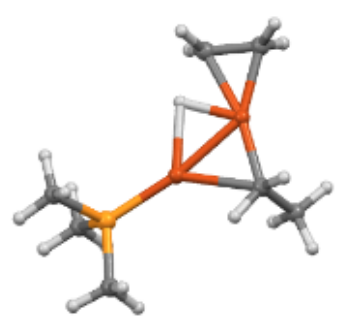

29

System = TS1CuI; C7H19Cu2P ; E(SCF) = -1014.25369334 Hartree; G(298.150 K) = -1014.064242 Hartree; Convergence on forces and displacements = 4 YES; Minimal frequency = -964.7253 cm-1; S**2 = None (restricted formalism)

Cu	2.798248	2.488696	0.156582
C	4.645977	3.357315	1.216971
Cu	4.669021	1.401826	0.925497
C	4.293303	-0.586594	0.448935
C	5.550626	-0.378169	1.156413
H	3.396856	0.596124	0.141352
H	4.350059	-0.882550	-0.598490
H	3.498281	-1.107003	0.982609
H	6.473514	-0.477765	0.589535
H	5.608017	-0.701130	2.193453
C	4.574741	3.689617	2.715036
H	5.326668	3.130507	3.282120
H	5.631801	3.619087	0.808582
H	3.971412	4.070726	0.688513
H	4.737669	4.759643	2.914805
P	0.848941	3.167755	-0.595673
C	0.502705	2.699536	-2.333805
H	1.256276	3.146878	-2.987098
H	-0.490718	3.043027	-2.639392
C	-0.580360	2.479959	0.323360
H	-0.548007	1.388432	0.278211
H	-1.524550	2.833940	-0.102464
H	-0.519429	2.785643	1.371015
H	0.605963	5.345842	0.445769
C	0.527516	4.973465	-0.578866
H	1.278530	5.480581	-1.190141
H	-0.469883	5.197609	-0.970672
H	3.599765	3.426663	3.140897
H	0.558306	1.612675	-2.433887

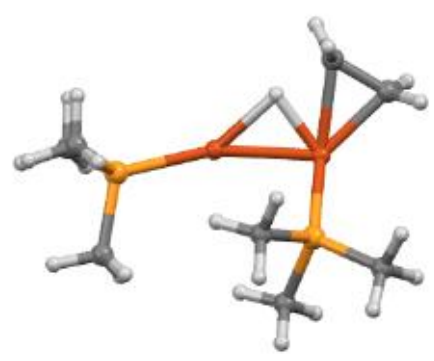

35

System = TS1Cu; C8H23Cu2P2(2) ; E(SCF) = -1396.09178398 Hartree; G(298.150 K) = -1395.857692 Hartree; Convergence on forces and displacements = 4 YES; Minimal frequency = -1000.4629 cm-1; S**2 = 0.7560

C	-0.530742	2.336099	0.881082
P	0.161810	0.901315	1.794138
Cu	-1.261820	0.048912	3.299431
Cu	-2.967960	-0.082980	4.944152
P	-3.268296	2.115245	4.898848
C	-3.950234	2.710015	3.301295
C	1.769952	1.549372	2.402389
C	0.662136	-0.235907	0.443748
C	-1.889054	-1.450114	6.246603
C	-3.317927	-1.369744	6.488770
C	-4.426078	2.877969	6.111996
C	-1.772809	3.173417	5.091512
H	-1.414619	-0.750234	5.021245
H	-1.492676	-2.397056	5.880655
H	-1.222118	-0.958377	6.955681
H	-3.926815	-2.234947	6.237112
H	-3.651737	-0.810880	7.360430
H	2.365299	1.962872	1.581483
H	1.588677	2.330563	3.145314
H	2.328247	0.741065	2.882001
H	1.309434	0.272477	-0.278164

H	1.194921	-1.091054	0.867981
H	-0.231295	-0.604814	-0.066742
H	0.181262	2.708055	0.137332
H	-1.452854	2.033316	0.378016
H	-0.767475	3.139133	1.584159
H	-4.085316	3.796777	3.304834
H	-3.266247	2.424734	2.497718
H	-4.911282	2.222612	3.116283
H	-2.016984	4.237391	4.998357
H	-1.324343	2.989680	6.071544
H	-1.042317	2.898054	4.326355
H	-4.526515	3.956974	5.950713
H	-5.406909	2.404601	6.015584
H	-4.056252	2.696793	7.124969

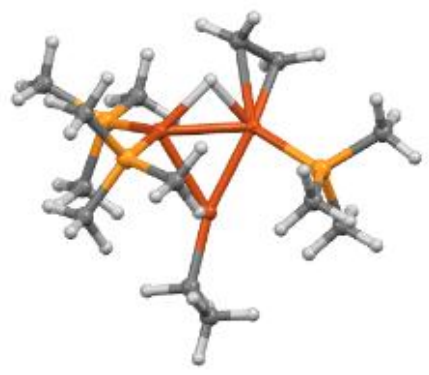

56

System = TS1_mix; C13H37Cu3P3(2) ; E(SCF) = -2133.82059777 Hartree; G(298.150 K) = -2133.426271 Hartree; Convergence on forces and displacements = 4 YES; Minimal frequency = -848.2280 cm-1; S**2 = 0.7580

Cu	0.692115	0.654358	2.519637
Cu	-1.056827	0.203783	0.858080
C	-2.740840	0.007122	-0.108253
H	-3.481305	-0.401718	0.598324
H	-2.599766	-0.765227	-0.880519
C	-3.276187	1.295022	-0.740792
H	-2.563967	1.722883	-1.458330
H	-4.224620	1.154254	-1.286949
H	-3.462236	2.069387	0.014400
P	-0.552060	2.464372	2.918446
C	0.028048	3.606906	4.244191
C	-0.913133	3.630041	1.544772
C	-2.238111	2.031629	3.501892
H	0.157503	3.041229	5.170746
H	0.996153	4.029089	3.960363
H	-0.684905	4.421246	4.414241
H	0.024336	4.030701	1.151224
H	-1.552331	4.454522	1.878784
H	-1.416373	3.079125	0.746246
H	-2.821062	2.930764	3.727132
H	-2.740328	1.451081	2.722316
H	-2.160770	1.410545	4.398302
P	1.437112	-2.465307	0.417921
C	2.716919	-3.455789	-0.465919
C	-0.118537	-3.226228	-0.196652
C	1.539654	-3.145167	2.121796
H	-0.959230	-2.784763	0.345010
H	-0.114048	-4.312926	-0.059262
H	-0.242039	-2.993861	-1.257696
H	3.709876	-3.167888	-0.108965
H	2.577221	-4.531272	-0.310018
H	2.661761	-3.238016	-1.536367
H	2.509578	-2.898383	2.561666
H	0.760192	-2.674546	2.726992
H	1.409658	-4.232639	2.127563
P	1.236176	0.763986	-1.741857
C	2.761692	0.836606	-2.769995
C	0.038051	-0.036423	-2.881035
C	0.673675	2.510940	-1.762749

H	-0.043384	0.508671	-3.827456
H	0.362451	-1.061296	-3.081354
H	-0.937877	-0.073542	-2.390688
H	0.606061	2.902332	-2.783402
H	-0.307280	2.563036	-1.285215
H	1.369078	3.122798	-1.182126
H	2.572356	1.318104	-3.735579
H	3.533766	1.396160	-2.234498
H	3.129774	-0.178975	-2.939839
Cu	1.282260	-0.221932	0.266379
C	1.880444	0.163590	4.167566
C	2.786331	0.261255	3.054363
H	1.633703	-0.821355	4.555285
H	1.838698	0.983004	4.879120
H	3.293105	-0.644637	2.726610
H	3.426683	1.141143	2.996492
H	2.172996	0.445353	1.726907

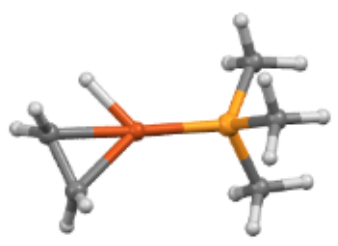

21

System = TS1; C5H14CuP ; E(SCF) = -737.622085273 Hartree; G(298.150 K) = -737.487122 Hartree; Convergence on forces and displacements = 3 YES; Minimal frequency = -885.4123 cm-1; S**2 = None (restricted formalism)

Cu	-1.595070	-0.689102	0.522301
C	0.264431	-1.469416	0.204719
H	0.692114	0.651342	0.522135
H	0.208390	0.207080	-1.198631
H	-1.037486	-2.186610	0.755397
C	0.220132	-0.064464	-0.146094
H	0.359521	-2.195284	-0.601367
H	0.833066	-1.758415	1.087191
P	-3.583323	0.079584	0.912673
C	-4.235533	1.261308	-0.329675
H	-4.290777	0.769285	-1.304376
H	-5.232032	1.616426	-0.047622
H	-3.552707	2.110724	-0.409819
C	-4.958211	-1.126404	1.072657
H	-4.730570	-1.824121	1.882464
H	-5.906069	-0.619604	1.281610
H	-5.049522	-1.695299	0.143612
C	-3.682643	1.023914	2.481673
H	-4.695603	1.408197	2.637253
H	-3.411420	0.372974	3.316719
H	-2.978609	1.858951	2.447618

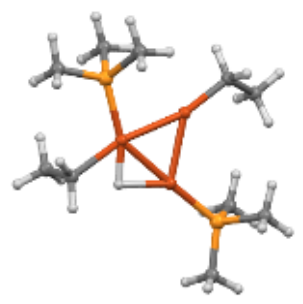

44

System = TS2CuI; C10H29Cu3P2 ; E(SCF) = -1673.36615002 Hartree; G(298.150 K) = -1673.066154 Hartree; Convergence on forces and displacements = 3 YES; Minimal frequency = -966.8257 cm-1; S**2 = None (restricted formalism)

Cu	1.440740	-0.926086	-0.196903
Cu	2.899597	-0.026739	1.467151
H	2.928578	-0.780073	-0.279722
Cu	0.513673	-0.171829	1.945236
C	2.673556	-1.249344	-1.890199
C	2.417479	-0.014020	-2.743083
H	3.742412	-1.479686	-1.839507
H	2.169750	-2.144494	-2.273434

H	1.349094	0.127909	-2.942699
H	2.927486	-0.057566	-3.716853
H	2.767129	0.896924	-2.239355
P	4.193106	0.879695	3.063969
C	6.013256	1.034608	2.877023
C	3.670219	2.590532	3.469913
C	4.013544	0.005253	4.667062
H	6.449759	0.042600	2.733478
H	6.236991	1.641514	1.995601
H	6.461392	1.501883	3.760139
H	3.843879	3.238305	2.606542
H	2.600287	2.592035	3.692602
H	4.222820	2.978143	4.331841
H	4.412530	-1.008463	4.575191
H	4.543195	0.532973	5.466843
H	2.951979	-0.065242	4.917853
P	-0.662780	-1.342436	-0.720234
C	-1.565363	-2.434661	0.441365
C	-1.705909	0.162441	-0.789191
C	-0.974248	-2.134556	-2.349225
H	-1.552690	-1.975315	1.434022
H	-1.058432	-3.401473	0.499709
H	-2.599287	-2.583967	0.114239
H	-2.737268	-0.087781	-1.056946
H	-1.296098	0.855011	-1.529097
H	-1.685913	0.647462	0.190585
H	-0.478182	-3.108599	-2.377711
H	-0.553643	-1.511751	-3.143220
H	-2.045793	-2.270448	-2.527286
C	-0.653230	0.299671	3.440843
H	-0.817813	-0.601462	4.055455
H	-1.644404	0.564761	3.034713
C	-0.143784	1.441425	4.330477
H	0.016002	2.362227	3.754405
H	-0.828666	1.700322	5.156287
H	0.820843	1.193354	4.791446

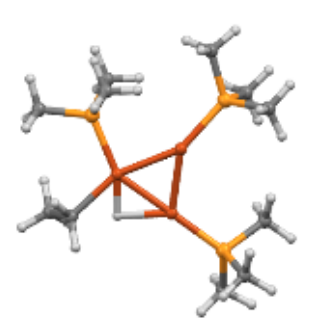

50

System = TS2Cu; C11H33Cu3P3(2) ; E(SCF) = -2055.21009375 Hartree; G(298.150 K) = -2054.864693 Hartree; Convergence on forces and displacements = 4 YES; Minimal frequency = -1112.9998 cm-1; S**2 = 0.7594

C	-0.118815	-1.966602	4.643835
P	-0.398104	-0.936905	3.146663
C	-2.106212	-1.424795	2.665062
Cu	1.270019	-0.995339	1.630420
Cu	3.476893	-0.065188	1.320972
P	4.760228	0.274121	3.105541
C	6.119851	1.516264	3.083010
Cu	2.136075	-0.496012	-0.562998
C	3.592738	-0.298131	-2.147651
C	3.425564	1.032244	-2.860707
C	-0.656267	0.735714	3.865542
C	5.603993	-1.198566	3.814790
C	3.734895	0.851744	4.518370
H	3.553108	0.124692	-0.541982
H	3.136495	-1.130610	-2.698460
H	4.645603	-0.547815	-1.985241
H	3.800333	1.858557	-2.242171
H	2.370370	1.247416	-3.064247

H	3.960644	1.070818	-3.821220
H	6.601686	1.602507	4.063252
H	6.865305	1.221506	2.339218
H	5.712233	2.488259	2.792372
H	6.122808	-0.950684	4.747381
H	4.859456	-1.975936	4.005122
H	6.327019	-1.585785	3.091632
H	4.343853	1.004097	5.415698
H	3.242973	1.790536	4.250292
H	2.964247	0.104555	4.723591
H	-1.421736	0.721096	4.648817
H	0.286627	1.093755	4.287084
H	-0.959199	1.426741	3.074500
H	-2.798474	-1.314485	3.506499
H	-2.449681	-0.801511	1.835564
H	-2.108342	-2.467485	2.335994
H	-0.925651	-1.838279	5.373266
H	-0.057958	-3.018439	4.351454
H	0.832463	-1.685294	5.103175
P	0.108813	-0.732370	-1.418433
C	-0.091214	-0.739116	-3.249195
C	-0.714534	-2.305854	-0.949200
C	-1.122878	0.530195	-0.902712
H	-2.117340	0.313422	-1.308401
H	-1.163663	0.552293	0.188820
H	-0.793959	1.513795	-1.248307
H	-1.736253	-2.354699	-1.340326
H	-0.133072	-3.144677	-1.340844
H	-0.731543	-2.386456	0.140917
H	-1.131331	-0.924946	-3.537438
H	0.226435	0.224918	-3.654597
H	0.545391	-1.517770	-3.678623

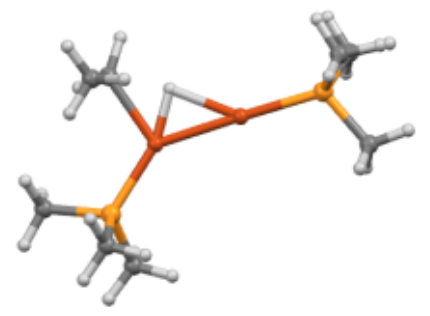

36

System = TS2; C8H24Cu2P2 ; E(SCF) = -1396.71229075 Hartree; G(298.150 K) = -1396.470066 Hartree; Convergence on forces and displacements = 2 YES; Minimal frequency = -1147.6997 cm-1; S**2 = None (restricted formalism)

C	4.699081	5.024807	4.482854
P	4.534686	4.328516	2.793289
C	4.292480	5.836953	1.767903
Cu	3.096168	2.702273	2.508824
Cu	2.517009	0.599270	3.448882
C	2.027758	2.833199	0.613659
C	3.117497	2.405297	-0.359613
C	6.256195	3.845027	2.379257
H	2.042155	1.809696	1.914811
H	1.850565	3.915558	0.609582
H	1.072656	2.346486	0.385410
H	3.978315	3.083805	-0.321619
H	2.782158	2.364969	-1.407126
H	3.499505	1.402300	-0.112089
H	4.978313	4.223836	5.172001
H	5.455624	5.816018	4.511937
H	3.736417	5.433393	4.801948
H	6.297263	3.504006	1.341351
H	6.562849	3.019154	3.025780
H	6.942603	4.687772	2.510859
H	5.091164	6.568274	1.929804

H 3.330370 6.292268 2.018503
H 4.270913 5.553216 0.712005
P 1.966284 -1.401010 4.416691
C 2.894746 -1.784877 5.955592
C 0.217681 -1.584414 4.953544
C 2.231976 -2.913227 3.405929
H 2.585459 -2.746743 6.377805
H 3.965181 -1.815134 5.734425
H 2.718866 -0.994781 6.690742
H 1.959151 -3.816039 3.962617
H 1.626341 -2.852824 2.497583
H 3.284014 -2.973739 3.114219
H 0.048106 -2.554743 5.432113
H -0.029917 -0.786355 5.658815
H -0.439636 -1.491144 4.084790

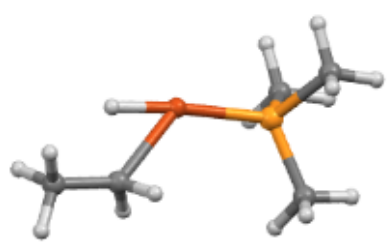

22
System = TS3; C5H15CuP(2) ; E(SCF) = -738.203770054 Hartree; G(298.150 K) = -738.062884 Hartree; Convergence on forces and displacements = 4 YES; Minimal frequency = -1095.9117 cm-1; S**2 = 0.7574
Cu 2.313356 3.329802 -1.249225
H 2.629788 4.760819 -1.626434
C 3.385318 4.932145 -0.172433
H 2.718082 4.825393 0.689875
H 4.271320 4.297481 -0.061625
C 3.721404 6.376165 -0.491294
H 4.334941 6.452641 -1.396507
H 2.814803 6.968794 -0.660288
H 4.278923 6.852255 0.326076
P 1.951248 1.273109 -0.529648
C 2.533727 0.858307 1.163184
C 2.727058 -0.030587 -1.558997
C 0.185210 0.783289 -0.484037
H 2.046059 1.517769 1.885950
H 3.613517 1.017714 1.223856
H 2.307465 -0.183136 1.414088
H 3.810653 0.113338 -1.572318
H 2.354798 0.047504 -2.583485
H 2.499121 -1.025158 -1.162807
H 0.076932 -0.249107 -0.136813
H -0.242254 0.877594 -1.485308
H -0.360003 1.449947 0.189302

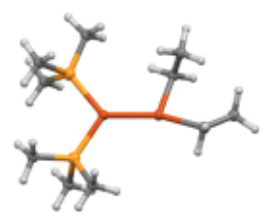

42
System = TS_but; C10H28Cu2P2 ; E(SCF) = -1475.28812700 Hartree; G(298.150 K) = -1474.991224 Hartree; Convergence on forces and displacements = 3 YES; Minimal frequency = -213.7840 cm-1; S**2 = None (restricted formalism)
Cu 0.482019 -0.431463 -0.154020
Cu 2.251526 -0.426390 1.438765
C 3.580624 -1.079082 2.781571
C 4.649207 -0.419206 3.614641
H 3.997777 -1.764990 2.022253
H 2.826094 -1.602990 3.390222
H 4.219815 0.311142 4.312254
H 5.217400 -1.137417 4.224759
H 5.376421 0.111214 2.987599
C 2.550851 1.320892 2.193665

C	1.625432	1.531838	3.370546
H	3.600580	1.483945	2.436720
H	2.267692	1.918134	1.324859
H	1.699120	2.571890	3.722550
H	0.581779	1.345059	3.099625
H	1.879500	0.881804	4.213313
P	-0.104845	-2.193893	-1.481982
C	1.157559	-2.401371	-2.805491
C	-0.084980	-3.846127	-0.672773
C	-1.666808	-2.300814	-2.464957
H	0.952737	-3.286937	-3.416677
H	1.159753	-1.514624	-3.445772
H	2.145307	-2.495300	-2.346832
H	-1.713408	-3.221061	-3.058656
H	-2.525613	-2.269213	-1.788407
H	-1.728176	-1.441005	-3.138320
H	-0.231389	-4.653300	-1.399010
H	0.874276	-3.977465	-0.165209
H	-0.877443	-3.890812	0.080006
P	-0.736896	1.384254	-0.835383
C	-2.571388	1.282167	-0.990255
C	-0.545443	2.939551	0.130435
C	-0.249717	1.938721	-2.522927
H	-1.112603	3.765951	-0.312171
H	-0.889802	2.773191	1.154971
H	0.513455	3.209357	0.168227
H	-0.472621	1.145821	-3.242206
H	-0.781152	2.850694	-2.815759
H	0.827189	2.127661	-2.544511
H	-2.999570	2.225321	-1.347804
H	-2.830549	0.481800	-1.688067
H	-3.004677	1.039556	-0.015731
